# Osmathiazole Ring: Extrapolation of an Aromatic Purely Organic System to Organometallic Chemistry

**DOI:** 10.1021/acs.organomet.2c00631

**Published:** 2023-02-09

**Authors:** María L. Buil, Miguel A. Esteruelas, Enrique Oñate, Nieves R. Picazo

**Affiliations:** Departamento de Química Inorgánica, Instituto de Síntesis Química y Catálisis Homogénea (ISQCH), Centro de Innovación en Química Avanzada (ORFEO-CINQA), Universidad de Zaragoza-CSIC, 50009 Zaragoza, Spain

## Abstract

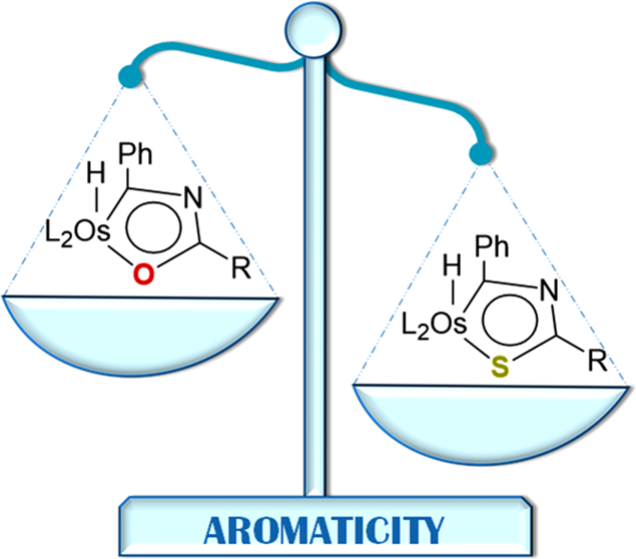

An osmathiazole skeleton has been generated starting from the cation of the salt [OsH(OH)(≡CPh)(IPr)(P^i^Pr_3_)]OTf (**1**; IPr = 1,3-bis(2,6-diisopropylphenyl)imidazolylidene; OTf = CF_3_SO_3_) and thioacetamide; its aromaticity degree was compared with that of thiazole, and its aromatic reactivity was confirmed through a reaction with phenylacetylene. Salt **1** reacts with the thioamide to initially afford the synthetic intermediate [OsH{κ^2^-*N,S*-[NHC(CH_3_)S]}(≡CPh)(IPr)(P^i^Pr_3_)]OTf (**2**). Thioamidate and alkylidyne ligands of **2** couple in acetonitrile at 70 °C, forming a 1:1 mixture of the salts [OsH{κ^2^-*C,S*-[C(Ph)NHC(CH_3_)S]}(CH_3_CN)(IPr)(P^i^Pr_3_)]OTf (**3**) and [Os{κ^2^-*C,S*-[CH(Ph)NHC(CH_3_)S]}(CH_3_CN)_3_(IPr)]OTf (**4**). Treatment of **3** with potassium *tert*-butoxide produces the NH-deprotonation of its five-membered ring and gives OsH{κ^2^-*C,S*-[C(Ph)NC(CH_3_)S]}(IPr)(P^i^Pr_3_) (**5**). The osmathiazole ring of **5** is slightly less aromatic than the osmathiazolium cycle of **3** and the purely organic thiazole. However, it is more aromatic than related osmaoxazoles and osmaoxazoliums. There are significant differences in behavior between **3** and **5** toward phenylacetylene. In acetonitrile, the cation of **3** loses the phosphine and adds the alkyne to afford [Os{η^3^-*C*_3_*,*κ^1^*-S*-[CH_2_C(Ph)C(Ph)NHC(CH_3_)S]}(CH_3_CN)_2_(IPr)]OTf (**6**), bearing a functionalized allyl ligand. In contrast, the osmathiazole ring of **5** undergoes a vicarious nucleophilic substitution of hydride, by acetylide, via the dihydride OsH_2_(C≡CPh){κ^2^-*C,S*-[C(Ph)NC(CH_3_)S]}(IPr)(P^i^Pr_3_) (**7**), which releases H_2_ to yield Os(C≡CPh){κ^2^-*C,S*-[C(Ph)NC(CH_3_)S]}(IPr)(P^i^Pr_3_) (**8**).

## Introduction

**Scheme 1:**
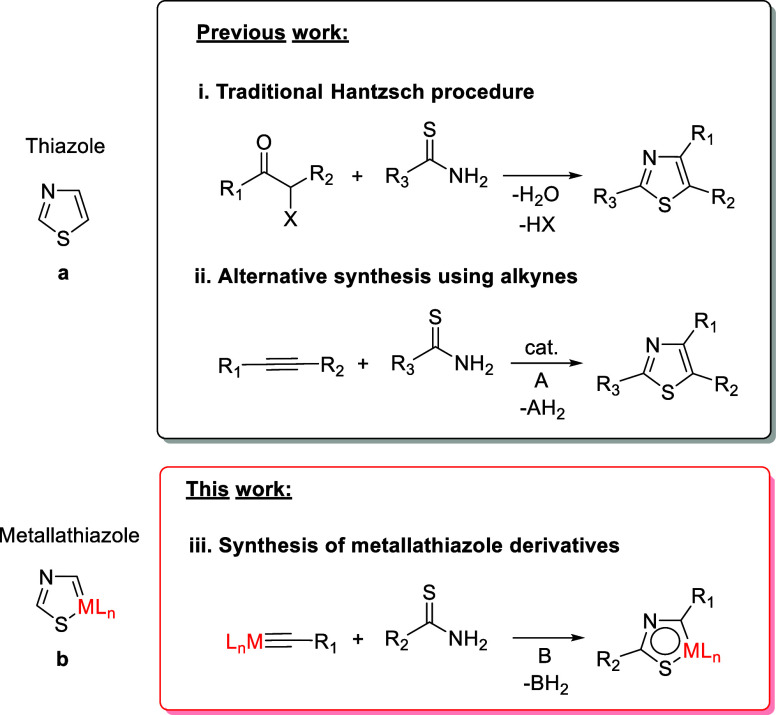
Synthetic Procedures for the Formation of Thiazole-Type Rings Using Thioamides

Thiazole is an aromatic five-membered diheteromonocycle posing sulfur and nitrogen at the 1- and 3-positions (**a** in Scheme 1). The thiazole ring is planar; its aromaticity is greater than that of its lighter oxygen counterpart, oxazole. The π-electron density distribution is concentrated on the heteroatoms. Thus, a thiazole molecule undergoes protonation at the nitrogen atom to afford thiazolium salts; its Brønsted basicity is about 3 times greater than that of the oxazole.^1^ The thiazole skeleton, which is found in a wide variety of natural products, forms essential parts of medicinally relevant compounds with applications as antimicrobial, anticancer, antitubercular, antioxidant, and anti-inflammatory agents, among other therapeutic activities.^2^ It is therefore one of the most significant structural components of pharmaceuticals; a recent analysis of the database of U.S. FDA approved drugs revealed that is in the top 25, being the most common five-membered aromatic N-heterocycle.^3^ A Hantzsch synthesis is a flexible traditional access to the thiazole skeleton. It involves the cyclocondensation of α-halogenocarbonyl compounds with thioamides (Scheme 1i).^4^ A variety of alkynes have been also used as alternatives to the α-halogenocarbonyls, keeping the thioamide as the source of the heteroatoms, but the presence of a catalyst is necessary in this case to lower the activation energy of the condensation (Scheme 1ii). The catalyst can be an acid,^5^ a transition metal,^6^ or a photocatalyst.^7^

**Scheme 2:**
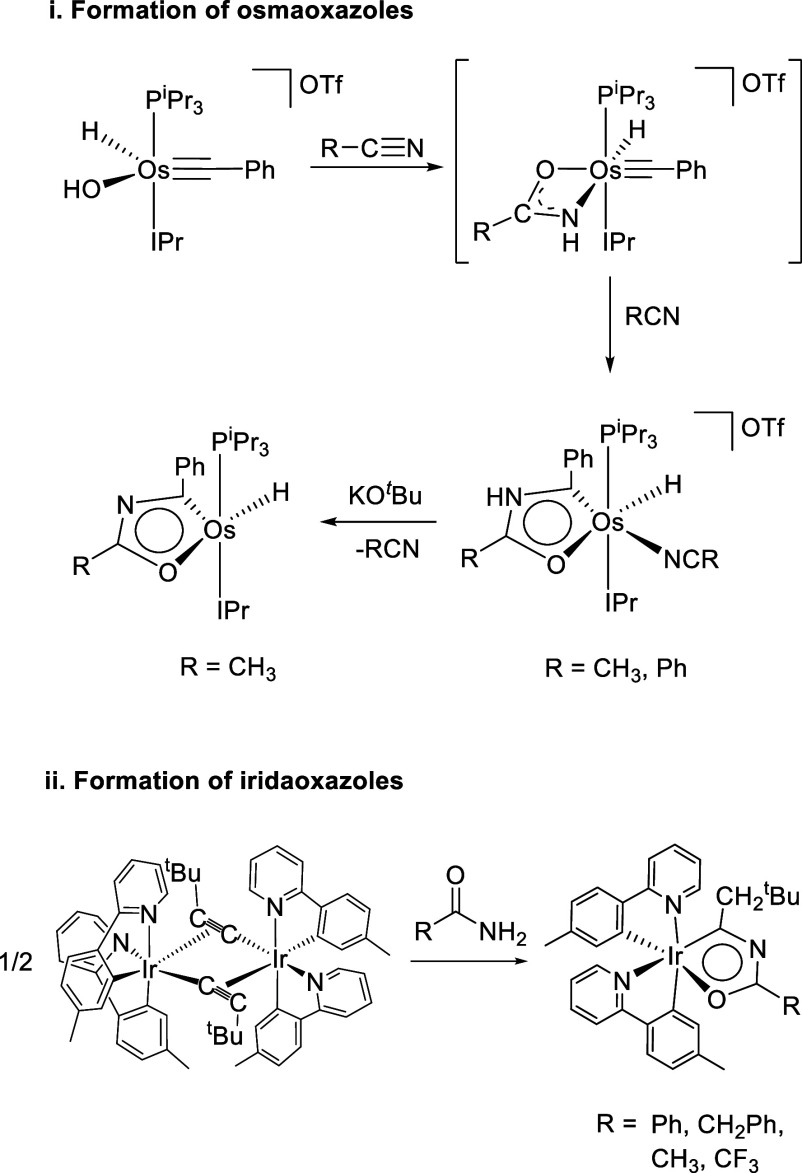
Synthetic Procedures for the Preparation of Metallaoxazoles

A fascinating conceptual challenge is to modify the properties of the aromatic organic molecules by means of the introduction of transition-metal features, to obtain organometallic reactivity. A promising approach toward a solution of the issue is to design synthetic strategies, based on organic precedents, which allow generating new aromatic organometallic entities. These species formally result from replacing a CH unit of the organic molecule by an isolobal metal fragment, formed by a transition metal and its associated ligands. Such a process performed on a thiazole should lead to a metallathiazole (b in Scheme 1). The existence of aromatic metallacycles was predicted by Thorn and Hoffmann in 1979;^8^ the hypothesis was confirmed by Roper and co-workers in 1982, with the preparation of the first osmabenzene.^9^ During the last 40 years, the aromaticity in organometallic cycles has been a topic in effervescence,^10^ which has experienced a tremendous development, reaching a notable degree of maturity from a conceptual point of view.^11^ Most of the effort has been focused on aromatic metallahydrocarbons,^12^ while mono- and polycyclic metallaheteroaromatic compounds have received comparatively less attention.^13^ The first monocycles containing two main-group heteroatoms were discovered very recently. They are the osmaoxazolium cations [OsH{κ^2^-*C,O*-[C(Ph)NHC(R)O]}(NCR)(IPr)(P^i^Pr_3_)]^+^, which afford the corresponding osmaoxazole molecules OsH{κ^2^-*C,O*-[C(Ph)NC(R)O]}(IPr)(P^i^Pr_3_), by deprotonation of the aromatic five-membered ring. The formation of the aromatic monocycle, containing both nitrogen and oxygen, takes place via transitory amidate species, which are generated by reaction between the hydroxide group of cation [OsH(OH)(≡CPh)(IPr)(P^i^Pr_3_)]^+^ and an external nitrile molecule, RC≡N. Once the amidates are generated on the metal coordination sphere, they cyclize with the alkylidyne ligand (Scheme 2i).^14^ The utility of amides in the preparation of this class of aromatic rings was strongly promoted a few months ago with the synthesis of the first iridaoxazole derivatives, Ir{κ^2^-*C,O*-[C(CH_2_^t^Bu)NC(R)O]}{κ^2^-*C,N*-(MeC_6_H_3_-py)}_2_, which were generated by the direct addition of these reagents to dimer *cis*-[Ir(μ_2_-η^2^-C≡CR){κ^2^-*C*,*N*-(MeC_6_H_3_-py)}_2_]_2_ (Scheme 2ii).^15^

The amidate–alkylidyne cyclization shown in Scheme 2i resembles the reactions summarized by the equation in Scheme 1ii. Such a similarity led us to see the metal–alkylidyne bond of the cation [OsH(OH)(≡CPh)(IPr)(P^i^Pr_3_)]^+^ as a synthon of a C–C triple bond of an alkyne, so that said cation should be able to be used to develop the organometallic version of the reactions shown in Scheme 1ii and, in this way, we would access the first metallathiazole. The success of such a hypothesis should also allow us to compare for the first time the degree of aromaticity of osmathiazoles and osmaoxazoles, from a theoretical point of view, but with experimental support.

This paper reports the preparation (Scheme 1iii) and full characterization of the first metallathiazolium cation and metallathiazole molecule and a comparative study of the aromaticity degree of these novel five-membered rings with those of their oxygen counterparts.

## Results and Discussion

### Osmathiazole Synthesis

**Scheme 3:**
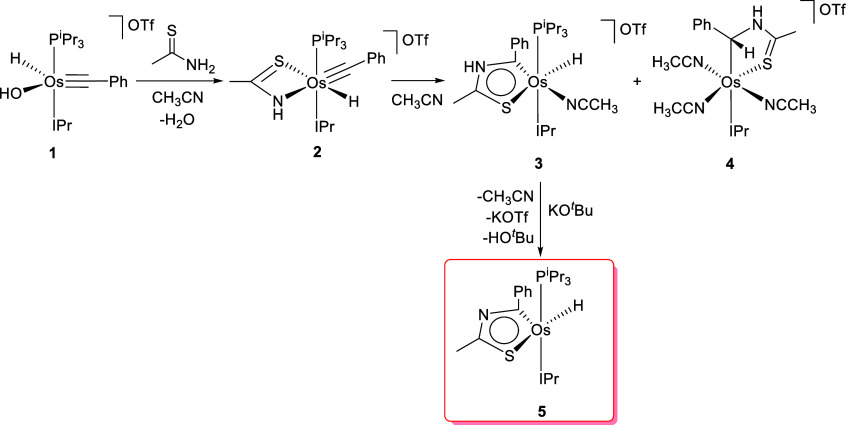
Osmathiazole Preparation

Our hypothesis was correct; the equation in Scheme 1ii has an organometallic version. As shown in Scheme 3 and summarized in the equation in Scheme 1iii, thioamides are useful reagents for forming osmathiazoles by N-addition to an osmium–alkylidyne bond, which acts as a synthon of the C–C triple bond of an alkyne. Thus, the preparation of osmathiazoles resembles that of osmaoxazoles, in the same way that many procedures for the preparation of thiazoles are closely related to some applied in the synthesis of their oxazole homologues, although there are also notable particularities that should be taken into account. Such peculiarities are mainly associated with the differences between oxygen and sulfur.

**Figure 1:**
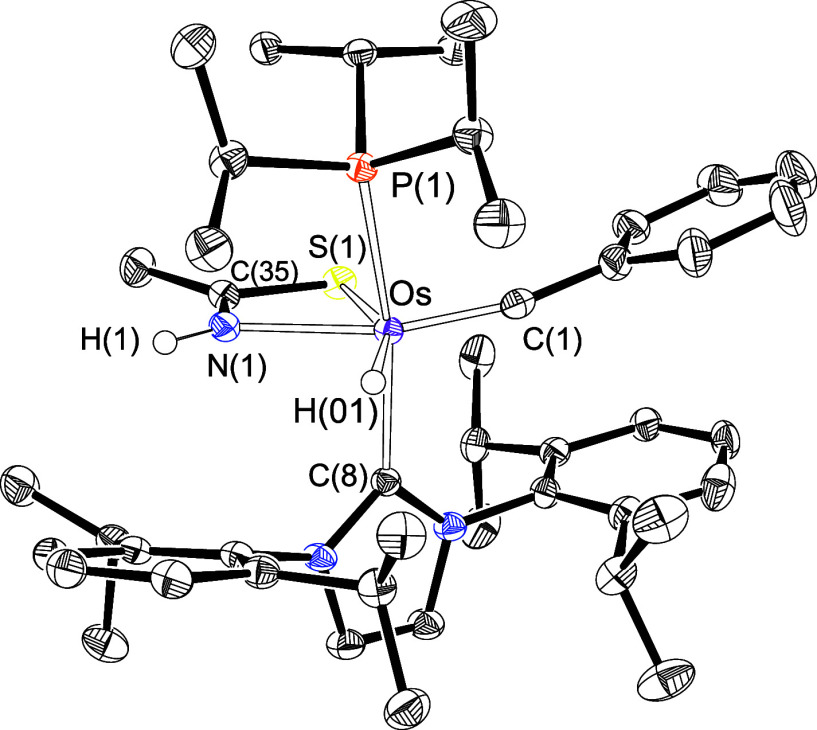
X-ray structure of complex **2** (ellipsoids shown at 50% probability). All hydrogen atoms are omitted for clarity (except OsH and NH). Selected bond distances (Å) and angles (deg): Os–P(1) = 2.405(10), Os–C(1) = 1.741(4), Os–C(8) = 2.111(4), Os–S(1) = 2.4958(9), Os–N(1) = 2.204(3), P(1)–Os–C(8) = 164.33(10), N(1)–Os–C(1) = 161.03(15), C(8)–Os–S(1) = 89.55(10), N(1)–Os–S(1) = 64.35(9).

The hydroxide ligand of the cation [OsH(OH)(≡CPh)(IPr)(P^i^Pr_3_)]^+^ is a strong enough Brønsted base to promote the deprotonation of the NH_2_ group of thioamides. Thus, the treatment of its trifluorosulfonate salt (**1**) with 1.0 equiv of thioacetamide, in acetonitrile, at room temperature produces the instantaneous replacement of the hydroxide ligand by a thioacetamidate group, to afford the corresponding six-coordinate cation. In contrast to the amidate counterpart proposed in Scheme 2i, this species is sufficiently stable to be isolated as the red salt [OsH{κ^2^-*N,S*-[NHC(CH_3_)S]}(≡CPh)(IPr)(P^i^Pr_3_)]OTf (**2**) in 83% yield. Complex **2** was characterized by an X-ray diffraction analysis. Figure 1 shows a view of the coordination polyhedron around the osmium center of the cation, which can be idealized as a distorted octahedron with the bulky phosphine and NHC ligands situated mutually *trans* (P(1)–Os–C(8) = 164.33(10)°). The most noticeable feature of the structure is the *trans* disposition of the NH group of the thioamidate ligand to the osmium-alkylidyne bond (N(1)–Os–C(1) = 161.03(15)°), in the plane perpendicular to the P(1)–Os–C(8) direction. Such a disposition explains why this compound can be isolated, since a rearrangement in the coordination sphere of the metal center is required to situate the NH group *cis* to the alkylidyne ligand, before a thioamidate–alkylidyne coupling takes place. The reason for the observed disposition is undoubtedly the poor π-donor ability of the sulfur atom, significantly lower than that of both oxygen and NH. It is well-known that the alkylidyne ligand directs the strongest π-donor coligand of the metal coordination sphere to its *trans* position, in this class of six-coordinate osmium(II)–alkylidyne complexes.^10e,16^ The structure shown in Figure 1 is consistent with the NMR spectra of the obtained red solid, in acetonitrile-*d*_3_, at room temperature. Notable features are a doublet (^2^*J*_H–P_ = 19.8 Hz) at −3.27 ppm in the ^1^H spectrum, corresponding to the hydride ligand, a singlet at 22.7 ppm in the ^31^P{^1^H} spectrum, due to the phosphine, and a doublet (^2^*J*_C–P_ = 10.1 Hz) at 275.9 ppm in the ^13^C{^1^H} spectrum, assigned to the alkylidyne C_α_ atom.

The activation energy for the thioamidate–alkylidyne coupling can be thermally overcome. As a consequence, a catalyst is not necessary to reach the coupling, in contrast to the purely organic version of the process. The rearrangement of ligands in the octahedral osmium coordination sphere appears to be possible because the phosphine dissociation from **2** is accessible under the reaction conditions. In agreement with this, heating of solutions of **2** in acetonitrile at 70 °C for 3 h afforded a mixture in about a 1:1 molar ratio of the expected osmathiazolium derivative [OsH{κ^2^-*C,S*-[C(Ph)NHC(CH_3_)S]}(CH_3_CN)(IPr)(P^i^Pr_3_)]OTf (**3**) and the free-phosphine tris(solvento) complex [Os{κ^2^-*C,S*-[CH(Ph)NHC(CH_3_)S]}(CH_3_CN)_3_(IPr)]OTf (**4**). Salts of the mixture were separated by fractional crystallization in dichloromethane–diethyl ether.

**Figure 2:**
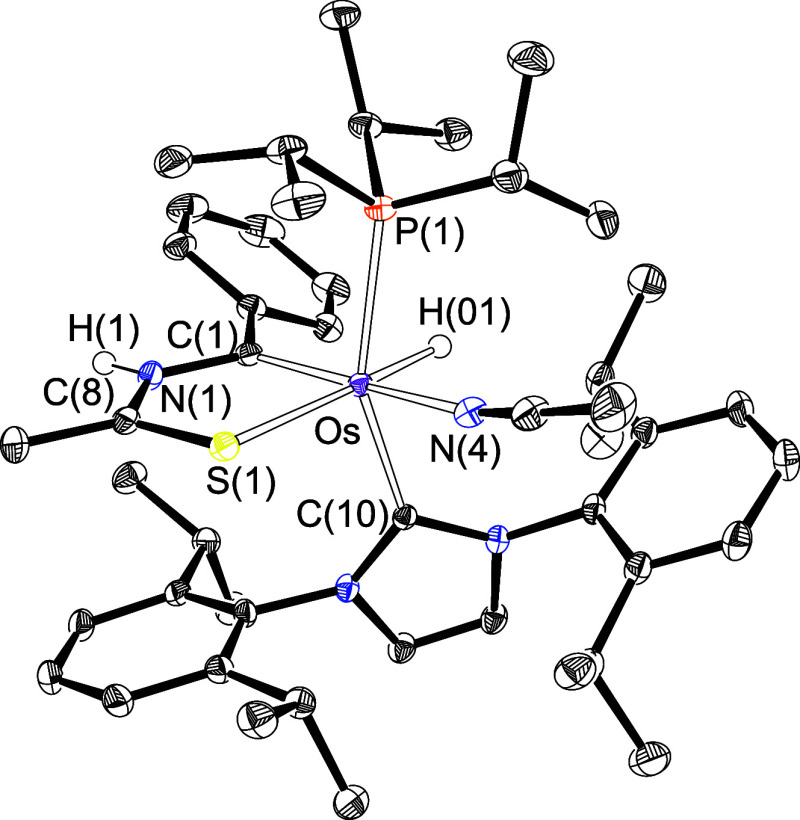
X-ray structure of complex **3** (ellipsoids shown at 50% probability). All hydrogen atoms are omitted for clarity (except OsH and NH). Selected bond distances (Å) and angles (deg): Os–P(1) = 2.3918(5), Os–C(1) = 1.957(2), Os–C(10) = 2.1118(19), Os–S(1) = 2.3457(5), Os–H(01) = 1.53(3), P(1)–Os–C(10) = 153.46(5), C(1)–Os–N(4) = 170.49(8), S(1)–Os–H(01) = 176.7(11), C(1)–Os–C(10) = 97.27(8), C(1)–Os–P(1) = 91.94(6).

Complex **3** was isolated as blue crystals in about 40% yield and characterized by an X-ray diffraction analysis. Figure 2 gives a view of the cation. The structure proves the migration of the NH-thioamidate to the alkylidyne carbon atom, to form the five-membered metalladiheteromonocycle, the metal center retaining its electronic saturation by means of the coordination of an acetonitrile molecule. The coordination around the osmium atom is the expected octahedral with the phosphine and NHC ligands situated in *trans* positions (P(1)–Os–C(10) = 153.46(5)°). The metallacycle lies perpendicular to an ideal P(1)–Os–C(10) direction with the C(1) atom situated *trans* to the acetonitrile molecule (C(1)–Os–N(4) = 170.49(8)°), while the sulfur atom is located *trans* to the hydride ligand (S(1)–Os–H(01) = 176.7(11)°). The NMR spectra of the blue crystals in acetonitrile-*d*_3_ at room temperature support the structure shown in Figure 2. The most noticeable resonance in the ^1^H spectrum is a broad singlet at 11.25 ppm, corresponding to the NH proton of the osmathiazolium ring, whereas the signal due to the hydride ligand appears at −7.95 ppm as a doublet with a P–H coupling constant of 26.5 Hz. In accord with the presence of the phosphine ligand in the cation, the ^31^P{^1^H} spectrum contains a singlet at 24.8 ppm. In the ^13^C{^1^H} spectrum, the C(1) atom gives rise to a doublet (^2^*J*_C–P_ = 2.7 Hz) at 223.4 ppm, while the signal corresponding to C(8) is observed at 192.9 ppm as a singlet.

**Figure 3:**
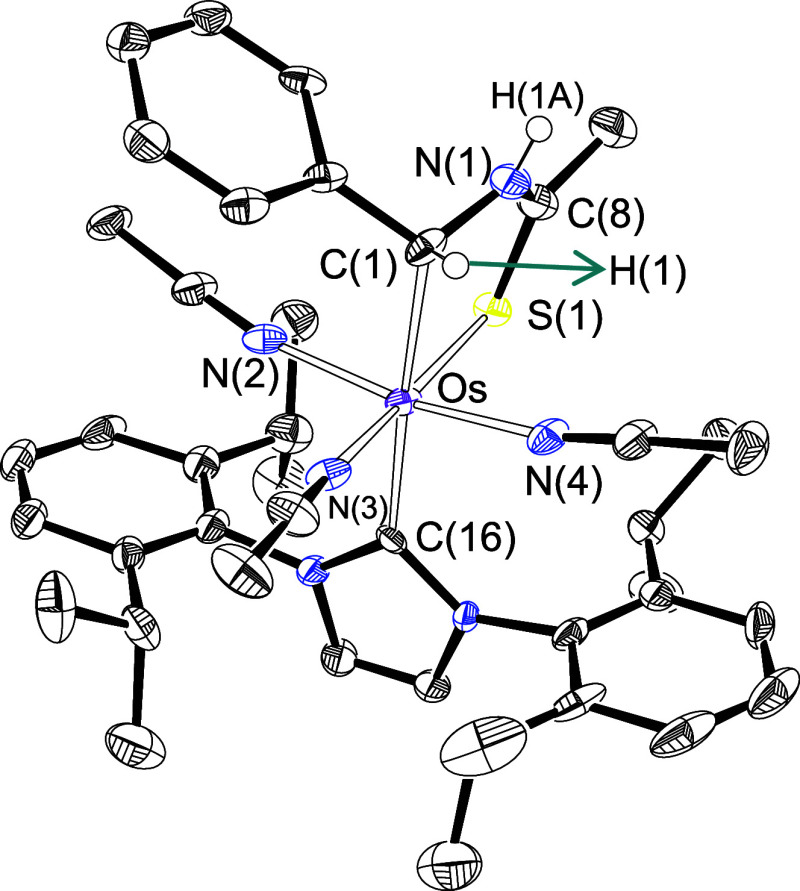
X-ray structure of complex **4** (ellipsoids shown at 50% probability). All hydrogen atoms are omitted for clarity (except H(1) and NH). Selected bond distances (Å) and angles (deg): Os–C(1) = 2.189(11), 2.197(10); Os–C(16) = 2.125(11), 2.103(10); Os–S(1) = 2.335(3), 2.336(3); Os–N(3) = 2.011(11), 2.050(8); C(1)–Os–C(16) = 179.1(4), 176.7(4); C(1)–Os–N(4) = 86.8(4), 81.9(4); S(1)–Os–N(3) = 173.4(3), 173.0(2).

Complex **4** was also isolated in about 40% yield and characterized by an X-ray diffraction analysis. The structure (Figure 3) demonstrates that in this case the five-membered ring results from the addition of both the hydride ligand and amidate group to the alkylidyne carbon atom, although it is not possible to assert which is added first. As a consequence of the double addition, the hybridization of the carbon atom changes to sp^3^. Consistently, the Os–C(1) distance increases by about 0.23 Å with regard to **3** (2.189(11) and 2.197(10) Å versus 1.9566(14) Å), whereas the resonance corresponding to this atom in the ^13^C{^1^H} NMR spectrum shifts about 160 ppm toward higher field, to appear at 64.5 ppm. One should in principle anticipate the transformation of **4** into **3** in the presence of triisopropylphosphine, due to the expected aromatic character of the osmathiazolium ring and therefore the higher stability of the latter. However, such a transformation does not occur in tetrahydrofuran at 40 °C. The kinetic inertia toward the dissociation of the acetonitrile ligands, *mer* disposed, in an octahedral environment around an osmium(II) center could be at the origin of this absence of reactivity,^17^ in view of the saturated character of the metal center. On the other hand, in spite of the mutually *cis* disposition of the hydride ligand and the C(1) atom of the osmathiazolium in **3**, the transformation of the latter into **4** does not takes place, even in acetonitrile at 70 °C for 48 h. Triisopropylphosphine protects the osmathiazolium character of the five-membered ring; it is clear that the release of the basic triisopropylphosphine from the osmium coordination sphere destabilizes the aromatic structure of the metalladiheterocycle, most probably as a consequence of the diminished basicity of the metal center. The decrease of electron density at osmium reduces its back-bonding ability to the carbon. This appears to generate a noticeable electron deficiency on such an atom, increasing its susceptibility to undergo the attack of nucleophiles.^15^

**Figure 4:**
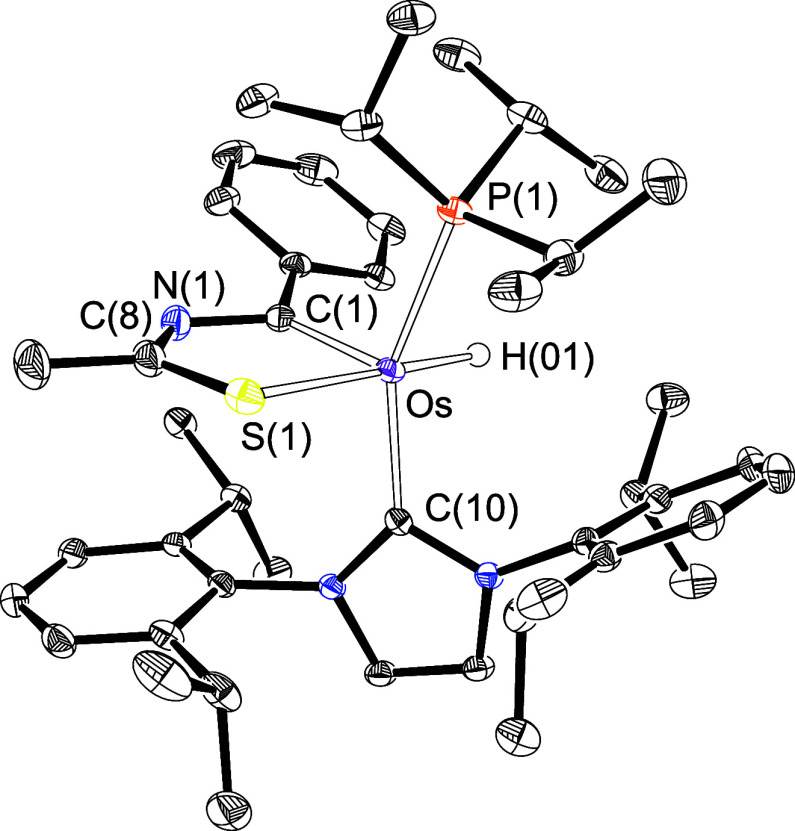
X-ray structure of complex **5** (ellipsoids shown at 50% probability). All hydrogen atoms are omitted for clarity (except OsH). Selected bond distances (Å) and angles (deg): Os–P(1) = 2.3471(5), Os–C(1) = 1.9145(19), Os–C(10) = 2.0678(18), Os–S(1) = 2.3886(5), Os–H(01) = 1.591(9), H(01)–Os–S(1) = 177.2(8), C(10)–Os–P(1) = 155.74(5), P(1)–Os–S(1) = 97.120(17), C(10)–Os–S(1) = 95.77(5).

The osmathiazolium of **3** is a Brønsted acid, as is any purely organic thiazolium cation. Thus, the treatment of solutions of the salt in tetrahydrofuran with 1.0 equiv of potassium *tert*-butoxide at room temperature produces the instantaneous deprotonation of the NH hydrogen atom. The abstraction of the proton gives rise to an adjustment of the electron density of the five-membered ring. The adjustment strongly influences the basicity of the metal center, which must tune its electron density by dissociating the acetonitrile ligand. The resulting osmathiazole molecule OsH{κ^2^-*C,S*-[C(Ph)NC(CH_3_)S]}(IPr)(P^i^Pr_3_) (**5**) was isolated as a purple solid in 72% yield and characterized by an X-ray diffraction analysis. Figure 4 gives a view of the structure. The coordination around the osmium(II) center can be idealized as a square pyramid with the C(1) atom of the metalladiheterocycle at the apex. The base is defined by the bulky monodentate ligands located in *trans* positions (C(10)–Os–P(1) = 155.74(5)°) and the hydride and sulfur atom of the five-membered ring, also in *trans* positions (H(01)–Os-S(1) = 177.2(8)°). The NMR spectra of the purple solid in toluene-*d*_8_ at room temperature are consistent with the structure shown in Figure 4. As expected for the presence of the hydride ligand, the ^1^H spectrum contains a doublet (^2^*J*_H–P_ = 15.3 Hz) at −3.08 ppm. The ^31^P{^1^H} spectrum shows a singlet at 43.5 ppm, resulting from the phosphine ligand. In the ^13^C{^1^H} spectrum, the most notable features are the resonances due to the C(1) and C(8) atoms of the metalladiheterocycle, at 228.8 and 208.1 ppm, respectively.

### Comparative Analysis of Some Parameters of the Metalladiheterocycles of **3** and **5** Related to Their Aromaticity

**Chart 1:**
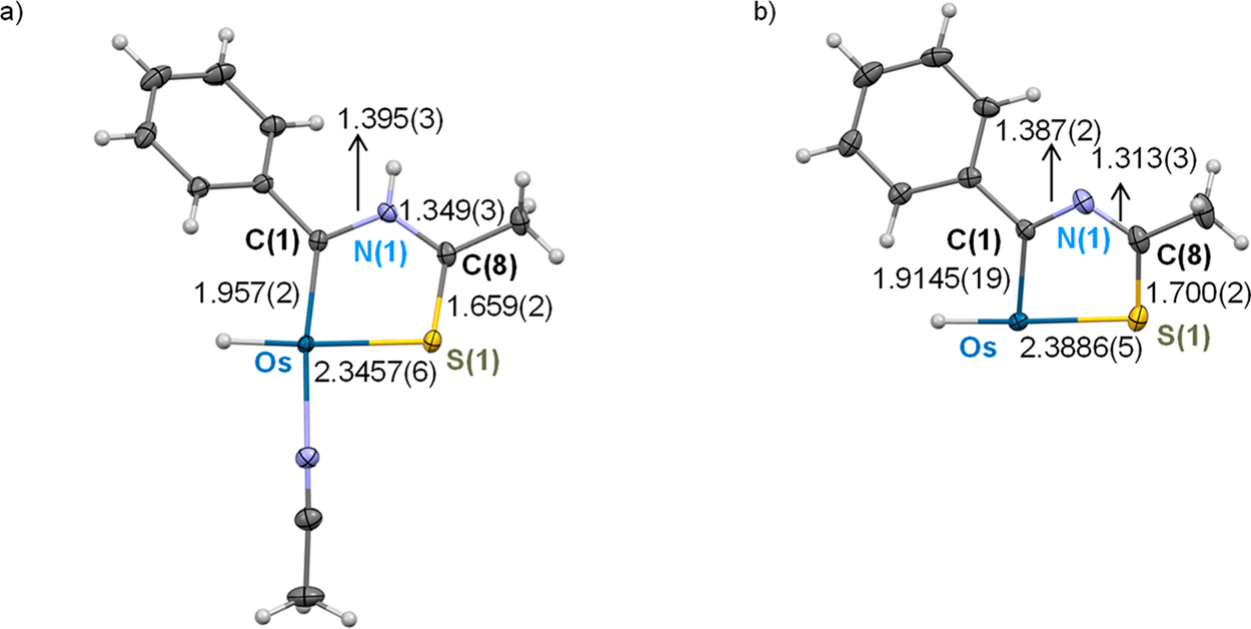
X-ray Bond Lengths (Å) in the Metalladiheterocycle of Complexes **3** (a) and **5** (b)

Complexes **3** and **5** are certainly new cases of aromatic organometallic heterocycles. The metalladiheterocycle of both compounds is planar. The maximum deviation from the best plane through the atoms Os, C(1), N(1), C(8), and S(1) involves C(1) and displays extremely small values of 0.0087(14) Å for **3** and 0.0353(15) Å for **5**. Furthermore, the bond lengths in the sequence Os–C(1)–N(1)–C(8)–S(1) are intermediate between the corresponding lengths of single and double bonds (Chart 1), in accordance with that expected for aromatic rings. The aromaticity of the metalladiheterocycles is also supported by the method of anisotropy of the induced current density (AICD), which clearly shows the occurrence of a diatropic (clockwise vectors) ring current within the ring (Figures S25 and S26), and the respective scans of the nuclear independent chemical shift (Figures S28 and S29), which provide NICS and NICS_*zz*_ values^18^ at the center of the ring and out of the plane at 1 Å above and below of −7.03, −7.79, and −8.86 (NICS) ppm and −2.04, −22.74, and −21.33 (NICS_*zz*_) ppm for **3** and −4.40, −4.88, and −7.59 (NICS) ppm and 6.06, −12.15, and −17.36 (NICS_*zz*_) ppm for **5**, respectively. These values compare well with those obtained for thiazole (−13.67, −11.44, and −11.44 (NICS) ppm and −10.85, −28.02, and −28.02 (NICS_*zz*_) ppm) and indicate that the replacement of the CH unit at the 5-position of the purely organic compound by the osmium atom and its coordinated ligands slightly reduces the aromaticity of the five-membered ring. They also suggest that the aromaticity degree in the osmathiazolium ring is higher than that in the osmathiazole cycle. An additional comparison of the NICS values obtained for the osmathiazolium and osmathiazole rings with those previously reported for the osmaoxazolium and osmaoxazole counterparts (−5.1, – 5.2, and −7.5 ppm and −2.8, – 3.4, and −6.5 ppm, respectively)^14^ reveals that **3** and **5** are more aromatic than the respective oxazolium and oxazole derivatives, in the same manner as the aromaticity of thiazole is greater than that of oxazole.

**Scheme 4:**
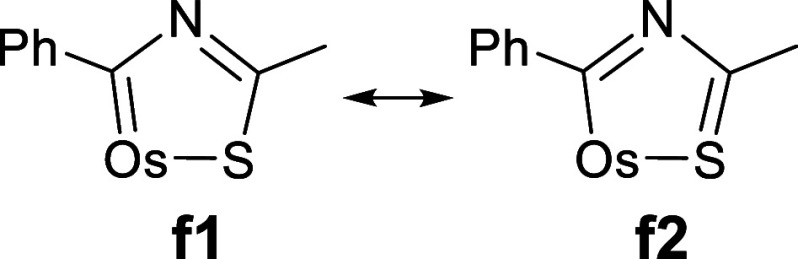
Resonance Forms Contributing to the Bonding Situation in the Osmathiazole Ring

The presence of distances intermediate between those corresponding to single and double bonds, for the bonds defining these five-membered rings, suggests that to describe the bonding situation in the metalladiheterocycles the f1 and f2 resonance forms must be taken into account (Scheme 4). To gain insight into which form makes a greater contribution to the structures, we conducted a natural bonding orbital (NBO) analysis on the bonding situation at **3** and **5**. In addition, we performed the same analysis for their lighter oxygen analogues and the purely organic molecules thiazole and oxazole for comparative purpouses.

**Chart 2:**
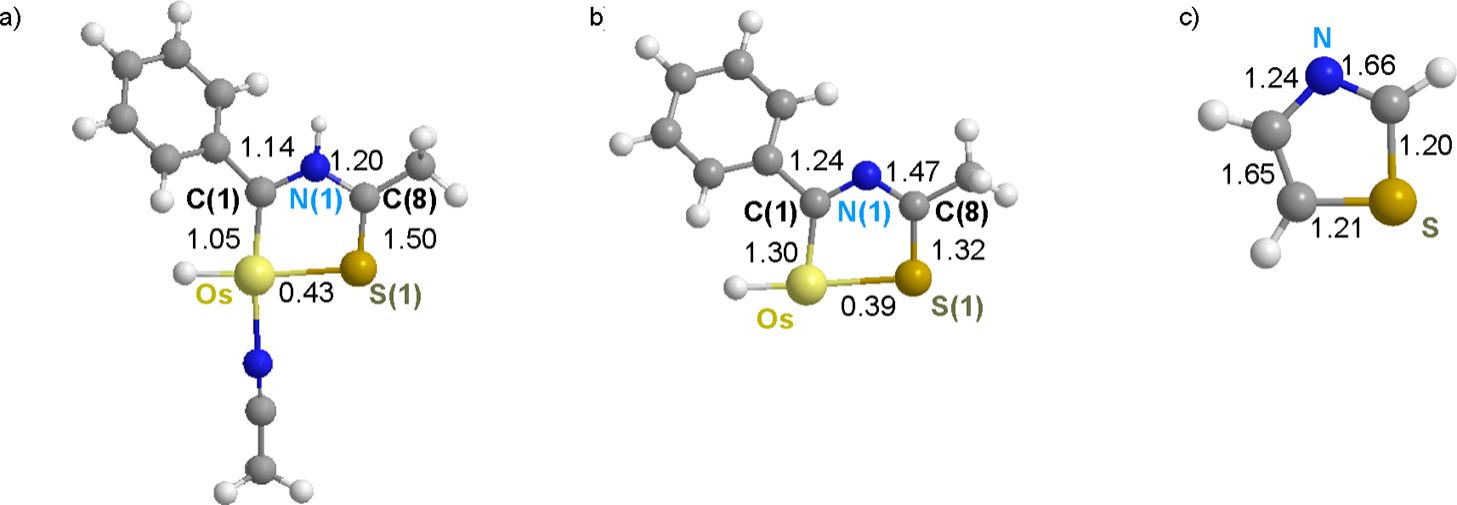
Wiberg Bond Indices for the Bonds of Osmathiazolium (a), Osmathiazole (b), and Thiazole (c)

The Wiberg bond indices are consistent with the existence of electron delocalization in the metalladiheterocycles. However, they also point out sites of electron concentration (Chart 2). The C(8)–S(1) bond of **3** is one of these places. Its highest Wiberg bond index of 1.50 suggests a predominant contribution of the f2 resonance form to the osmathiazolium. In contrast, the stronger bonds of **5** appear to be Os–C(1) and N(1)–C(8), with indices of 1.30 and 1.47, in accordance with a greater contribution of the f1 resonance form to osmathiazole. Resonance form f1 is also the main contribution to the thiazole structure. This is in agreement with the localization of the π-orbitals of the rings (Figures S32 and S33), which are situated between C(1) and N(1) and between C(8) and S(1) for **3** and between Os and C(1) and between N(1) and C(8) for **5**.

**Chart 3:**
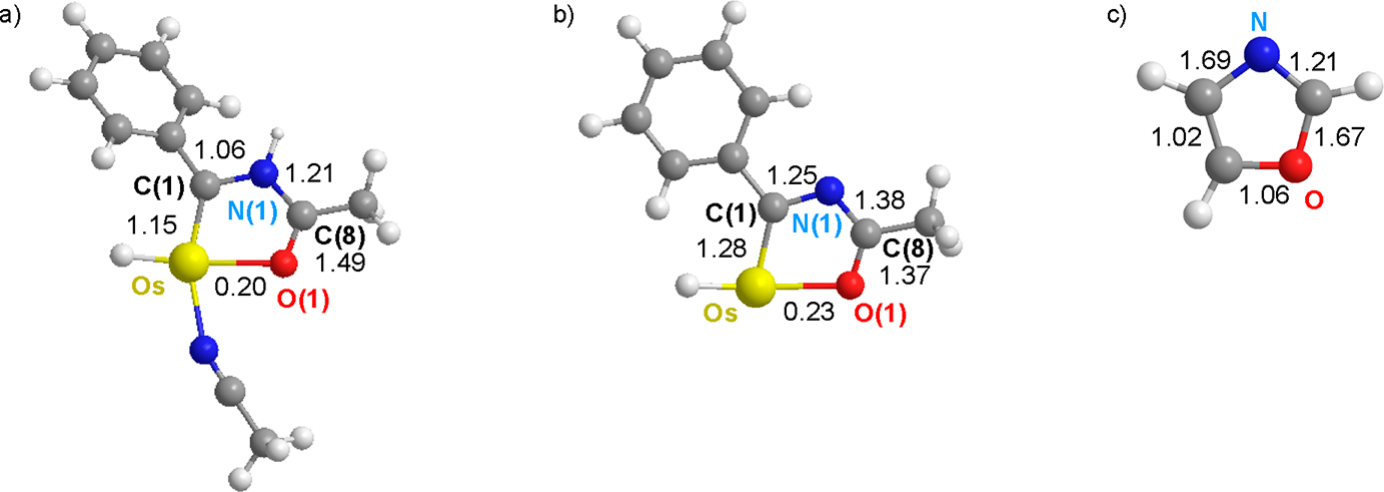
Wiberg Bond Indices for the Bonds of Osmaoxazolium (a), Osmaoxazole (b), and Oxazole (c)

Resonance form f2 makes a more significant contribution than f1 to the oxazole structure, in contrast to thiazole, according to the Wiberg bond indices for the bonds of the former. Nevertheless, the bonding situations in osmaoxazolium and osmaoxazole are similar to those in osmathiazolium and osmathiazole, f2 making the greater contribution to the osmaoxazolium and f1 the main contribution to the osmaoxazole (Chart 3).

An overview of the Wiberg bond indices indicates that replacement of the CH unit, at position 5 of the thiazole, with the osmium(II) fragment decreases the contribution of the f1 resonance form to the ring structure, since the difference between the Wiberg indices along the bond sequence of the metalladiheterocycle is mitigated. At the same time, the strength of the Os–S(1) bond decreases with respect to the strength of the bond between the carbon atom at position 5 and the sulfur in the thiazole. The same comparison in the case of the oxygen homologue reveals that substitution, in contrast, causes an increased contribution of f1 to the five-membered ring structure, while generating a weaker Os–O bond than the bond between the atom carbon at position 5 and the oxygen atom in the oxazole.

**Chart 4:**
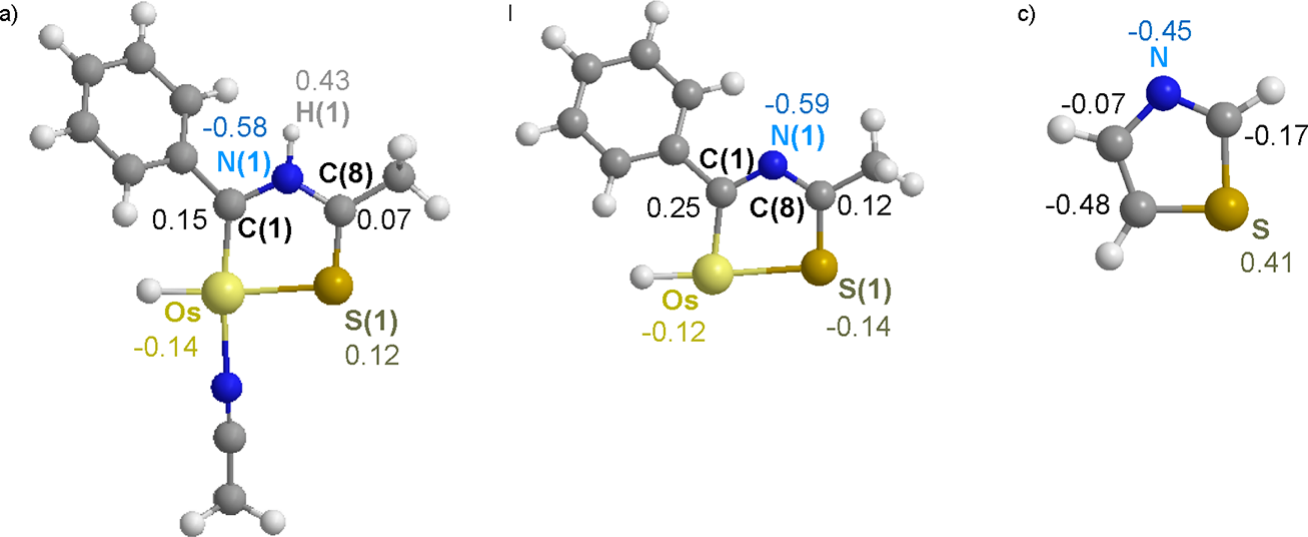
(a) NBO Charges on the Five-Membered-Ring Atoms of Osmathiazolium (a), Osmathiazole (b), and Thiazole (c)

The NBO charges on the five-membered-ring atoms are also dramatically affected by the CH-by-L_*n*_Os replacement in the thiazole, with the exception of the nitrogen atom; not only are the absolute values of the charges significantly different but also the signs (Chart 4).

**Chart 5:**
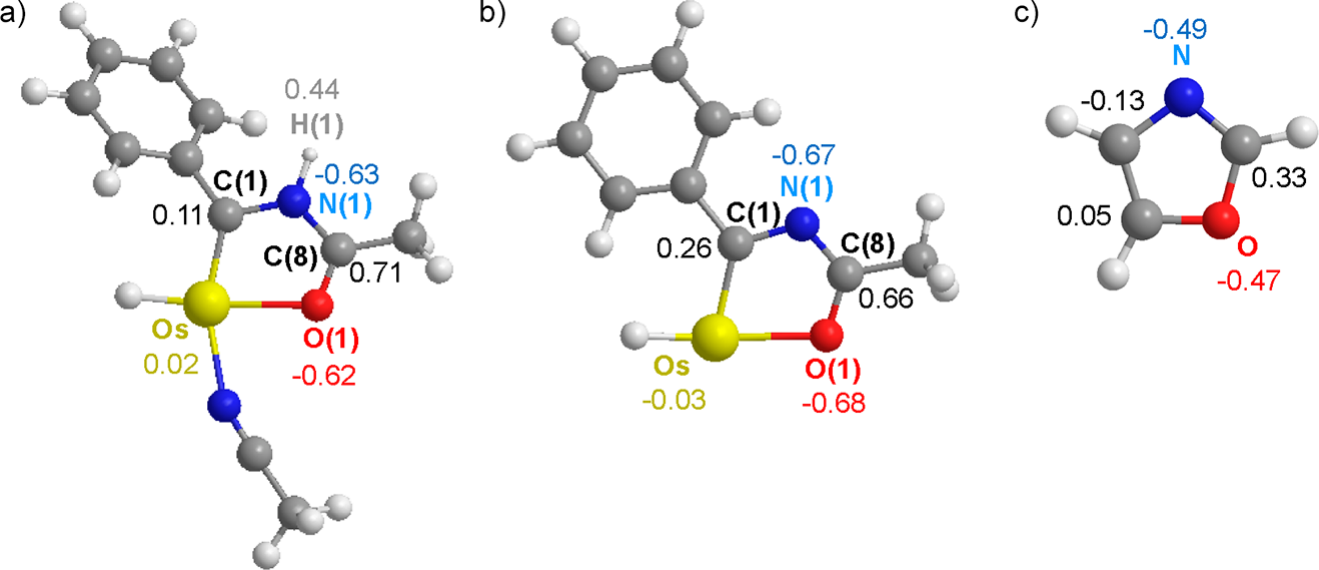
NBO Charges on the Five-Membered-Ring Atoms of Osmaoxazolium (a), Osmaoxazole (b), and Oxazole (c)

The disruption is significantly more moderate in the case of oxazole; only the carbon atom attached to the metal center of the osmaoxazole undergoes a sign change with respect to the carbon at the same position in the oxazole (Chart 5).

The deprotonation of **5** significantly disrupts the NBO charge of the sulfur atom, which passes from 0.12 in the osmathiazolium to −0.14 in the osmathiazole, in addition to causing the dissociation of the acetonitrile molecule from the osmium coordination sphere. In contrast to osmathiazolium, the deprotonation of osmaoxazolium alters the NBO charges of the atoms of the Os–C bond, which go from 0.02 and 0.11 in the cation to −0.03 and 0.26 in the molecular species.

### Reactions of **3** and **5** with Phenylacetylene

Although both **5** and **3** are aromatic species, their reactivities toward phenylacetylene are very different. The difference in behavior seems to be associated with marked variations in the lability of the phosphine, between the cation and the molecular derivative, and in the saturated or unsaturated nature of the metal center. The easy dissociation of triisopropylphosphine from the cation destabilizes the aromatic system, allowing nucleophile migration from the metal center to the neighboring carbon atom, as evidenced by the formation of **4**. On the other hand, the five-coordinate character of osmium(II) of the molecular derivative prevents the release of the phosphine and favors oxidative addition reactions, the system giving a typical aromatic reactivity that even includes the metal center.

**Scheme 5:**
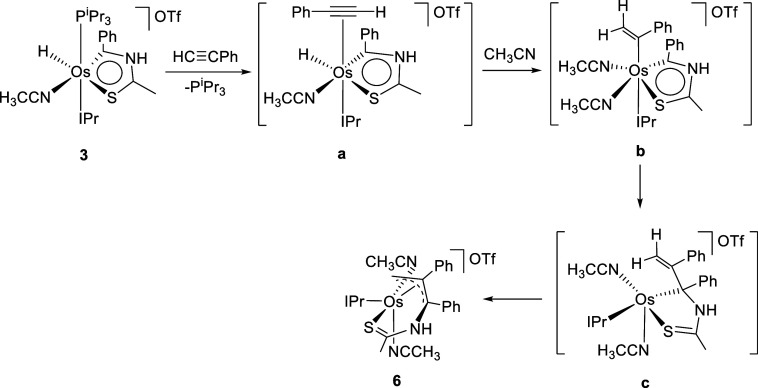
Formation of **6**

Treatment of solutions of **3** in acetonitrile with 1.1 equiv of phenylacetylene at room temperature for 1 h leads to [Os{η^3^-*C*_3_*,*κ^1^*-S*-[CH_2_C(Ph)C(Ph)NHC(CH_3_)S]}(CH_3_CN)_2_(IPr)]OTf (**6**). Its formation is the result of a Markóvnikov-type addition of the hydride ligand and the C(1) atom of the cation to the C–C triple bond of the alkyne, along with the substitution of triisopropylphosphine by a solvent molecule. This reaction leading to a functionalized allyl ligand should be highlighted, since it is a multicomponent organometallic synthesis on the osmium coordination sphere, involving an external organic molecule and three coordinated ligands of the thioamidate cation **2**. The process can be rationalized according to Scheme 5. Alkyne could initially displace the phosphine ligand, to afford the π-alkyne intermediate **a**. The subsequent insertion of the C–C triple bond into the Os–H bond should give the alkenyl species **b**, which could evolve by migration of such an alkenyl ligand to C(1) to form the σ-allyl intermediate **c**. Thus, the transformation of the σ-allyl moiety in π-allyl could finally yield **6**. The addition of an unsaturated organic fragment to aromatic metallacycles has previously given rise to interesting types of polycyclic derivatives^13a,19^ and 9- and 10-membered osmacycles.^20^

**Figure 5:**
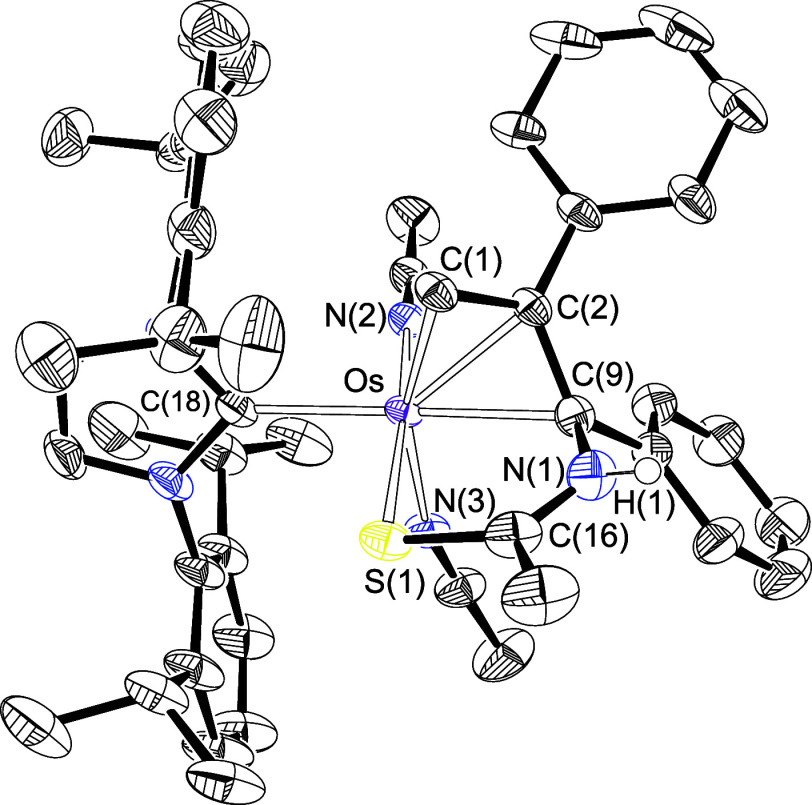
X-ray structure of complex **6** (ellipsoids shown at 50% probability). All hydrogen atoms are omitted for clarity (except NH). Selected bond distances (Å) and angles (deg): Os–C(1) = 2.161(4), Os–C(2) = 2.158(4), Os–C(9) = 2.199(4), Os–S(1) = 2.3453(10), Os–C(18) = 2.103(4), Os–N(2) = 2.028(3), Os–N(3) = 2.067(3), C(1)–C(2) = 1.443(5), C(2)–C(9) = 1.453(5), N(2)–Os–S(1) = 172.91(9), N(3)–Os–C(1) = 160.30(15), C(18)–Os–C(9) = 165.75(16), C(1)–C(2)–C(9) = 116.3(3).

Salt **6** was isolated as a yellow solid in 84% yield and characterized by an X-ray diffraction analysis. Figure 5 shows a view of the structure of the cation. The coordination around the osmium atom can be idealized as an octahedron. The functionalized allyl occupies one face, whereas the IPr and acetonitrile ligands lie at the other face. The skeleton of the tridentate group provides resonances at 198.0 (C(16)), 94.5 (C(9)), 79.4 (C(2)), and 29.8 (C(1)) ppm in the ^13^C{^1^H} NMR spectrum in dichloromethane-*d*_2_ at room temperature.

**Scheme 6:**
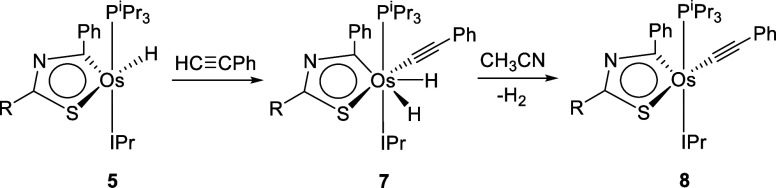
Vicarious Nucleophilic Substitution of Hydride by Acetylide at 5

The unsaturated character of **5** is certainly a key factor that determines its behavior toward phenylacetylene and marks the difference from **3**. This nature, along with the remarkable basicity of the osmium center, gives it the ability to oxidatively aggregate the C(sp)–H bond of the alkyne to form the osmium(IV)–dihydride OsH_2_(C≡CPh){κ^2^-*C,S*-[C(Ph)NC(CH_3_)S]}(IPr)(P^i^Pr_3_) (**7**). The addition was instantaneous in toluene as solvent. The dihydride was isolated as a brown solid in 44% yield (Scheme 6). In accordance with the presence of the hydride ligands, the ^1^H NMR spectrum in toluene-*d*_8_ at room temperature shows a doublet (^2^*J*_H–P_ = 15.7 Hz) at −7.89 ppm, which exhibits a 300 MHz *T*_1_(min) value of 97 ± 4 ms at 243 K. In the ^13^C{^1^H} NMR spectrum, the osmathiazole carbon atoms generate resonances at 272.4 (OsC) and 204.1 (NCS) ppm. The ^31^P{^1^H} NMR spectrum contains a singlet at 29.3 ppm, as a consequence of the phosphine presence. In acetonitrile, at room temperature, complex **7** undergoes the reductive elimination of molecular hydrogen to give the osmium(II) derivative Os(C≡CPh){κ^2^-*C,S*-[C(Ph)NC(CH_3_)S]}(IPr)(P^i^Pr_3_) (**8**). In toluene, under 1 atm of H_2_, complex **8** regenerates **7.** The overall process from **5** to **8** is a nice example of “*vicarious nucleophilic substitution of hydrogen*” occurring on an organometallic electron-deficient aromatic ring.^21^ Dihydride **7** is the intermediate σ^H^ adduct. In the same manner as observed for osmaoxazole molecules,^14^ but in contrast to classical organic reactions,^22^ a base is not necessary to promote the abstraction of the leaving group of the nucleophile, since the dihydride intermediate has the ability of H_2_ release.

**Figure 6:**
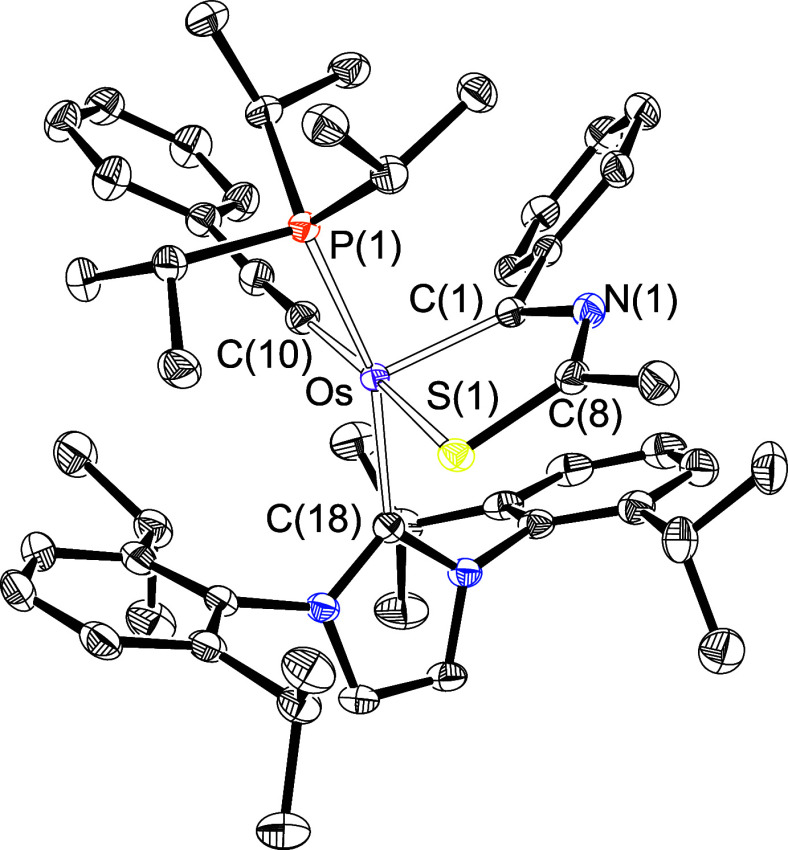
X-ray structure of complex **8** (ellipsoids shown at 50% probability). All hydrogen atoms are omitted for clarity. Selected bond distances (Å) and angles (deg): Os–P(1) = 2.3981(4), Os–C(1) = 1.9265(15), Os–C(10) = 2.0090(16), Os–C(18) = 2.1058(15), Os–S(1) = 2.3770(4), C(1)–Os–S(1) = 80.07(5), C(10)–Os–S(1) = 176.30(5), C(18)–Os–P(1) = 160.13(4), S(1)–Os–P(1) = 92.409(14).

Complex **8** was isolated as a purple solid in 53% yield and characterized by an X-ray diffraction analysis. Figure 6 offers a view of the structure, which resembles that of **5** with the acetylide in the hydride position and angles at the base of the pyramid of 176.30(5)° (C(10)–Os–S(1)) and 160.13(4)° (C(18)–Os–P(1)). Like its hydride counterpart, the osmathiazole ring is planar, with C(1) showing the maximum deviation from the best plane through its defining atoms (0.0935(9) Å). The NICS (−3.7, −5.54, and −6.05 ppm) and NICS_*zz*_ (10.2, −10.97, and −12.18 ppm) values of the diheterometallacycle compare well with those of **5**, although they also indicate an aromaticity slightly lower than that of the latter. Such a decrease appears to be related to a diminished basicity of the metal center as revealed by the NBO charges (−0.01 versus −0.12), which is consistent with the lower σ-donor power of the acetylide ligand.^23^ The reduction of the basicity of the osmium center decreases its back-bonding ability to C(1), which is manifested in the diminution of the Wiberg bond index of the Os–C(1) bond with respect to that of **5** (1.26 versus 1.30). As a result of the weakening of the back-bond, C(1) suffers from electron deficiency and its resonance in the ^13^C{^1^H} NMR spectrum is shifted to lower field by approximately 20 ppm with respect to the chemical shift observed in **5**, appearing now at 245.1 ppm. In this context, it should be noted that, according to the increase in electrophilicity of C(1), the NBO charge of this atom in **8** is 0.27 while in **5** it is 0.25. Signals at 130.6 (C_α_) and 115.7 (C_β_) ppm, corresponding to the alkynyl ligand, and a singlet at 21.4 ppm in the ^31^P{^1^H} NMR spectrum, due to the phosphine, are other features of **8**.

## Concluding Remarks

This study shows the discovery of a new class of organometallic heterocycle, metallathiazole: in particular, osmathiazole. The preparation procedure used is an extrapolation of a purely organic method for the synthesis of thiazoles to organometallic chemistry. The purely organic procedure involves the catalytic addition of a thioamide to the C–C triple bond of an alkyne. Now, taking the Os–C triple bond of an osmium–alkylidyne compound as an alkyne synthon, we have built the five-membered ring of the diheterometallacycle without the need for a catalyst through a sequence of three reactions: formation of an alkylidyne–osmium–thioamidate intermediate, thioamidate–alkylidyne coupling to form an osmathiazolium ring, and deprotonation of osmathiazolium.

An analysis of the aromaticity of the new diheterometallacycle on the basis of experimental structural data and DFT calculations, including the NBO method, reveals that the generated osmathiazole ring is slightly less aromatic than its protonated form osmathiazolium and purely organic thiazole. However, it is more aromatic than its related lighter oxygen counterpart, osmaoxazole and osmaoxazolium cycles. The coordinately unsaturated metal center of the osmathiazole ring displays a notable basicity, which gives it the ability to oxidatively aggregate σ-bonds, including the C(sp)–H bond of terminal alkynes. As a consequence, the aromatic osmathiazole ring is able to undergo a vicarious nucleophilic substitution of hydride by acetylide at the metal center. In contrast to the reactions performed with purely organic aromatic systems, a base is not necessary to promote the abstraction of the leaving group of the nucleophile, the acidic hydrogen atom of the terminal alkyne.

In summary, a new aromatic diheterometallacycle has been discovered, the degree of aromaticity provided has been compared with that of the pure organic equivalent, and its aromatic reactivity has been confirmed.

## Experimental Section

### General Information

All reactions were carried out exclusively under an atmosphere of argon, using Schlenk tube techniques. Only dried solvents were used. General details such as X-ray analysis, instrumental methods, and computational information can be found in the Supporting Information. Chemical shifts (expressed in ppm) in the NMR spectra (Figures S1–S23) are referenced to the residual solvent peaks, and coupling constants (*J*) are given in hertz. Signals were assigned through two-dimensional experiments (^1^H–^1^H COSY, ^1^H–^13^C{^1^H} HMBC, and ^1^H–^13^C{^1^H} HSQC). Starting complex **1** was synthesized as reported in a previous published method.^16c^

### Preparation of [OsH{κ^2^-*N,S*-[NHC(CH_3_)S]}(≡CPh)(IPr)(P^i^Pr_3_)]OTf (**2**)

Thioacetamide (8 mg, 0.11 mmol) was added to a solution of [OsH(OH)(≡CPh)(IPr)(P^i^Pr_3_)]OTf (**1**; 100 mg, 0.12 mmol) in 5 mL of acetonitrile at room temperature. The mixture changed from yellow to red. After stirring for 5 min, the solvent was removed *in vacuo* and diethyl ether was added to afford a red solid, which was repeatedly washed with diethyl ether (3 × 3 mL). Yield: 87 mg (83%). X-ray-quality crystals were grown by slow diffusion of pentane into a solution of **2** in tetrahydrofuran at 4 °C in a drybox. Anal. Calcd for C_46_H_67_F_3_N_3_O_3_OsPS_2_: C, 52.50; H, 6.42; N, 3.99; S, 6.09. Found: C, 52.83; H, 6.20; N, 4.34; S, 6.45. MS (electrospray, *m*/*z*): C_45_H_67_N_3_OsPS [M]^+^, 904.4302; found, 904.4374. IR (cm^–1^): ν(Os–H) 2159 (w); ν(NH) 1587 (w); ν(SO_3_) 1261 (s); ν(C = S) 1145 (w); ν(CF_3_) 1031 (s). ^1^H NMR (400 MHz, CD_3_CN, 298 K): δ 7.60 (m, 1H, Ph), 7.49 (s, 2H, *H*-IPr), 7.29 (d, ^3^*J*_H–P_ = 7.8, 2H, Ph), 7.20–7.12 (4H, Ph), 7.01 (d, ^3^*J*_H–P_ = 7.8, 2H, Ph), 6.92 (d, ^3^*J*_H–P_ = 8.0, 2H, Ph), 6.08 (br, 1H, N*H*), 3.33 (sept, ^3^*J*_H–H_ = 6.7, 2H, C*H*CH_3_), 2.52 (sept, ^3^*J*_H–H_ = 6.7, 2H, C*H*CH_3_), 2.26 (sept, ^3^*J*_H–H_ = 7.6, 3H, PC*H*CH_3_), 1.74 (s, 3H, C*H*_3_), 1.52 (d, ^3^*J*_H–H_ = 6.7, 6H, CHC*H*_3_), 1.23 (dd, ^2^*J*_H–P_ = 13.2, ^3^*J*_H–H_ = 7.1, 9H, PCHC*H*_3_), 1.24 (d, ^3^*J*_H–H_ = 6.7, 6H, CHC*H*_3_), 1.10 (d, ^3^*J*_H–H_ = 6.7, 12H, CHC*H*_3_), 0.85 (dd, ^2^*J*_H–P_ = 14.5, ^3^*J*_H–H_ = 7.1, 9H, PCHC*H*_3_), −3.27 (d, ^2^*J*_H–P_ = 19.8, 1H, Os–*H*). ^31^P{^1^H} NMR (121.4 MHz, CD_3_CN, 298 K): δ 22.7 (s). ^13^C NMR plus HMBC and HSQC (101 MHz, CD_3_CN, 298 K): δ 279.5 (d, ^2^*J*_C–P_ = 10.1, Os≡*C*), 203.5 (s, S*C*N), 174.1 (d, ^2^*J*_C–P_ = 78.1, N*C*N), 152.5 (s, C_*ipso*_–Ph), 147.6, 146.8, 146.5, and 138.0 (all s, C_*ipso*_–CPh + C_*o*_–CPh), 132.2, 131.4, 129.7, 129.0, 128.1, 127.6, 126.6, 124.6, 123.8, and 122.6 (all s, Ph + CPh), 125.4 (s, *C*–IPr), 35.8 (s, *C*H_3_), 30.3 and 29.6 (both s, *C*HCH_3_), 27.9 and 27.7 (both s, *C* HCH_3_), 26.5 (d, ^3^*J*_C–P_ = 18.4, P*C* HCH_3_), 23.3 and 22.6 (both s, CH*C*H_3_), 19.8 and 19.4 (both s, PCH*C*H_3_).

### Treatment of [OsH{κ^2^-*N,S*-[NHC(CH_3_)S]}(≡CPh)(IPr)(P^i^Pr_3_)]OTf (**2**) with Acetonitrile. Formation of [OsH{κ^2^-*C,S*-[C(Ph)NHC(CH_3_)S]}(CH_3_CN)(IPr)(P^i^Pr_3_)]OTf (**3**) and [Os{κ^2^-*C,S*-[CH(Ph)NHC(CH_3_)S]}(CH_3_CN)_3_(IPr)]OTf (**4**)

A red solution of **2** (100 mg, 0.09 mmol) in acetonitrile (5 mL) was stirred for 3 h at 70 °C. After this time, the solvent of the resulting green solution was removed under vacuum. The addition of 5 mL of diethyl ether gave rise to the precipitation of a pale green solid, which was washed with 3 × 7 mL of diethyl ether and dried under vacuum. ^1^H NMR spectrum shows a mixture of complexes **3** and **4** in a 1:1 molar ratio. The salts were separated by fractional crystallization. The green solid was dissolved in 5 mL of dichloromethane. Then, 8 mL of diethyl ether was slowly added without stirring. After 3 days, yellow crystals of complex **4** were formed. Yield: 42.8 mg (44%). The mother liquor was evaporated to dryness, and the residue was dissolved in 5 mL of dichloromethane. Then, 8 mL of pentane was slowly added without stirring. After 3 days, blue crystals of complex **3** were formed. Yield: 40.6 mg (39%).

#### Data for **4**

Anal. Calcd for C_43_H_55_F_3_N_6_O_3_OsS_2_: C, 50.87; H, 5.46; N, 8.28; S, 6.32. Found: C, 50.69; H, 5.48; N, 8.18; S, 5.98. MS (electrospray, *m*/*z*): C_40_H_52_N_5_OsS [M – CH_3_CN]^+^, 826.3553; found, 826.3552. IR (cm^–1^): ν(NH) 2243 (w); ν(SO_3_) 1281 (s); ν(C=S) 1150 (w); ν(CF_3_) 1033 (s). ^1^H NMR (400 MHz, CD_3_CN, 330 K): δ 8.50 (s, 1H, N*H*), 7.42–7.31 (6H, Ph), 7.08 (t, ^3^*J*_H–H_ = 7.6, 2H, Ph), 6.89 (t, ^3^*J*_H–H_ = 7.3, 1H, Ph), 6.88 (s, 2H, *H*-IPr), 6.61 (d, ^3^*J*_H–H_ = 7.2, 2H, Ph), 5.30 (s, 1H, OsC*H*), 3.06 (sept, ^3^*J*_H–H_ = 6.8, 4H, C*H*CH_3_), 2.39 (s, 3H, C*H*_*3*_), 1.35 (d, ^3^*J*_H–H_ = 6.8, 6H, CHC*H*_*3*_), 1.27 (d, ^3^*J*_H–H_ = 6.8, 6H, CHC*H*_*3*_), 1.15 (d, ^3^*J*_H–H_ = 6.8, 6H, CHC*H*_*3*_), 1.14 (d, ^3^*J*_H–H_ = 6.8, 6H, CHC*H*_*3*_). ^1^H NMR (400 MHz, CD_3_CN, 298 K, unobserved signals in aliphatic region at high temperature): δ 2.48 (s, 3H, N≡CC*H*_*3*_), 2.02 (s, 3H, N≡CC*H*_*3*_), 1.57 (s, 3H, N≡CC*H*_*3*_). ^13^C NMR plus HMBC and HSQC (101 MHz, CD_3_CN, 330 K): δ 201.2 (s, N*C*S), 170.0 (s, N*C*N), 153.0 (s, *C*_*ipso*_-Ph), 148.0, 147.9, and 140.14 (all s, *C*_*ipso*_-CPh + *C*_*o*_-CPh), 131.3, 128.4, 127.0, 124.9, 124.1, and 123.0 (all s, Ph + CPh + *C*-IPr), 119.1, 115.6, and 115.5 (all s, N≡*C*CH_3_), 64.5 (s, Os*C*H), 29.6 and 29.5 (both s, *C*HCH_3_), 25.9 (s, CH*C*H_3_), 23.7 (s, *C*H_3_), 23.6 (s, CH*C*H_3_), 4.9 and 4.8 (both s, N≡C*C*H_3_).

#### Data for **3**

Anal. Calcd for C_48_H_70_F_3_N_4_O_3_OsPS_2_: C, 52.73; H, 6.45; N, 5.12; S, 5.86. Found: C, 52.47; H, 6.06; N, 4.76; S, 5.67. MS (electrospray, *m*/*z*): C_45_H_67_N_3_OsPS [M – CH_3_CN]^+^, 904.4402; found, 904.4410. IR (cm^–1^): ν(Os–H) 2259 (w); ν(NH) 1541 (w); ν(SO_3_) 1250 (s); ν(C=S) 1145 (w); ν(CF_3_) 1028 (s). ^1^H NMR (400 MHz, CD_3_CN, 298 K): δ 11.25 (s, 1H, N*H*), 7.74 (d, ^3^*J*_H–H_ = 7.9, 2H, *o*-Ph), 7.74 (dd, ^3^*J*_H–H_ = 7.3, ^4^*J*_H–H_ = 1.4, 2H, Ph), 7.44 (t, ^3^*J*_H–H_ = 7.7, 2H, Ph), 7.37–7.31 (5H, Ph), 7.17 (br, 2H, Ph), 7.04 (br, 2H, *H*-IPr), 2.58 (s, 3H, S=CC*H*_*3*_), 2.45 (m, 2H, C*H* CH_3_), 1.96 (s, 3H, N≡CC*H*_*3*_), 1.70 (m, 3H, PC*H* CH_3_), 1.62 (d, ^3^*J*_H–H_ = 6.7, 6H, CHC*H*_*3*_), 1.09 (m, 6H, CHC*H*_*3*_), 0.96 (d, ^3^*J*_H–H_ = 6.7, 6H, CHC*H*_*3*_), 0.79 (dd, ^2^*J*_H–H_ = 13.4, ^3^*J*_H–H_ = 7.1, 9H, PCHC*H*_*3*_), 0.39 (dd, ^2^*J*_H–H_ = 13.4, ^3^*J*_H–H_ = 7.1, 9H, PCHC*H*_*3*_), −7.95 (d, ^2^*J*_H–H_ = 26.5, 1H, Os-*H*). ^1^H NMR (400 MHz, CD_3_CN, 263 K, unobserved signals in aliphatic region at room temperature): 4.03 and 2.77 (both m, 1H each, C*H* CH_3_), 1.37 and 1.24 (both d, ^3^*J*_H–H_ = 6.7, 3H each, CHC*H*_*3*_). ^31^P{^1^H} NMR (162 MHz, CD_3_CN, 298 K): δ 24.8 (s). ^13^C NMR plus HMBC and HSQC (101 MHz, CD_3_CN, 298 K): δ 223.4 (d, ^2^*J*_C–P_ = 2.7, Os*C*), 192.9 (s, N*C*S), 164.7 (d, ^2^*J*_C–P_ = 53.4, N*C*N), 154.7 (s, *C*_*ipso*_-Ph), 147.9 and 147.2 (both s, *C*_*ipso*_-CPh + *C*_*o*_-CPh), 130.9, 130.6, 129.9, 128.7, 126.9, and 124.3 (all s, Ph + CPh + *C*-IPr), 30.4 and 29.2 (both s, *C*HCH_3_), 26.8 (d, ^2^*J*_C–P_ = 25.4, P*C*HCH_3_), 26.2 (s, CH*C*H_3_), 24.1 (s, *C*H_3_), 22.6 (s, N≡C*C*H_3_), 18.6 and 18.4 (PCH*C*H_3_).

### Preparation of OsH{κ^2^-*C,S*-[C(Ph)NC(CH_3_)S]}(IPr)(P^i^Pr_3_) (**5**)

Potassium *tert*-butoxide (11 mg, 0.10 mmol) was added to a solution of [OsH{κ^2^*-S*,*C*-[CH_3_]NHC[Ph]}(N≡CCH_3_)(IPr)(P^i^Pr_3_)]OTf (**3**; 100 mg, 0.09 mmol) in THF (5 mL). After 5 min, at room temperature, the solvent was removed under vacuum and the residue was treated with toluene. The resulting suspension was filtered over Celite, and the solvent was evaporated to dryness. The addition of acetonitrile afforded a purple solid, which was decanted and washed three times with more acetonitrile (3 mL). Yield: 59 mg (72%). X-ray-quality crystals of **5** were obtained in pentane at 4 °C in a drybox. Anal. Calcd for C_45_H_66_N_3_OsPS: C, 59.90; H, 7.37; N, 4.66; S, 3.55. Found: C, 60.06; H, 7.54; N, 4.40; S, 3.85. MS (electrospray, *m*/*z*): C_45_H_67_N_3_OsPS [M + H]^+^, 904.4403; found, 904.4396. IR (cm^–1^): ν(Os–H) 1980 (w); ν(C=S) 1382 (w). ^1^H NMR (400 MHz, Tol-*d*_8_, 298 K): δ 8.49 (d, ^3^*J*_H–H_ = 7.8, 2H, *o*-Ph), 6.98–6.95 (3H, Ph), 6.92 (s, 1H, *H*-IPr), 6.90 (s, 1H, *H*-IPr), 6.88 (dd, ^3^*J*_H–P_ = 1.7, ^4^*J*_H–H_ = 7.8, 2H, Ph), 6.80 (d, ^3^*J*_H–H_ = 1.7, 1H, Ph), 6.78 (d, ^3^*J*_H–H_ = 1.7, 1H, Ph), 6.21 (s, 2H, *H*-IPr), 3.04 (sept, ^3^*J*_H–H_ = 6.7, 2H, C*H*CH_3_), 2.88 (m, 2H, C*H*CH_3_), 2.85 (s, 3H, S=CC*H*_*3*_), 1.56 (d, ^3^*J*_H–H_ = 6.8, 6H, CHC*H*_*3*_), 1.53 (sept, ^3^*J*_H–H_ = 8.3, 3H, PC*H*CH_3_), 1.09 (d, ^3^*J*_H–H_ = 6.8, 3H, CHC*H*_*3*_), 0.91 (d, ^3^*J*_H–H_ = 6.8, 6H, CHC*H*_*3*_), 0.85 (d, ^3^*J*_H–H_ = 6.8, 6H, CHC*H*_*3*_), 0.55 (dd, ^2^*J*_H–P_ = 12.7, ^3^*J*_H–H_ = 7.2, 9H, PCHC*H*_*3*_), 0.54 (d, ^3^*J*_H–H_ = 6.8, 3H, CHC*H*_*3*_), 0.37 (dd, ^2^*J*_H–P_ = 12.7, ^3^*J*_H–H_ = 7.2, 9H, PCHC*H*_*3*_). ^1^H NMR (400 MHz, Tol-*d*_8_, 213 K, high-field region): δ −3.08 (d, ^2^*J*_H–P_ = 15.3, 1H, Os-*H*). ^31^P {^1^H} NMR (162 MHz, Tol-*d*_8_, 298 K): δ 43.5 (s). ^13^C NMR plus HMBC and HSQC (101 MHz, Tol-*d*_8_, 298 K): δ 228.8 (Os*C*, inferred from the HMBC spectrum), 208.1 (br, N*C*S), 186.8 (N*C*N, inferred from the HMBC spectrum), 155.4 (s, *C*_*ipso*_-Ph), 146.8, 146.7, and 136.7 (all s, *C*_*ipso*_–CPh + *C*_*o*_-CPh), 131.5, 129.8, 126.5, 126.2, 123.9, and 123.7 (all s, Ph + CPh + *C*-IPr), 29.3, 28.8, 26.5, and 26.3 (all s, *C*HCH_3_), 25.9 (d, ^3^*J*_C–P_ = 25.4, P*C*HCH_3_), 23.5 and 23.1 (both s, CH*C*H_3_), 19.4 and 19.0 (both s, PCH*C*H_3_).

### Preparation of [Os{η^3^-*C*_3_*,*κ^1^*-S*-[CH_2_C(Ph)C(Ph)NHC(CH_3_)S]}(CH_3_CN)_2_(IPr)]OTf (**6**)

Phenylacetylene (11.0 μL, 0.10 mmol) was added to a blue solution of complex **3** (100 mg, 0.09 mmol) in 5 mL of acetonitrile. The mixture was stirred for 1 h at room temperature. After that, the resulting yellow solution was concentrated under vacuum and 5 mL of diethyl ether was added. A yellow solid appeared which was washed with more diethyl ether (3 × 5 mL). Yield: 84 mg (85%). X-ray-quality crystals were grown by slow diffusion of diethyl ether into a solution of **6** in dichloromethane at 4 °C in a drybox. Anal. Calcd for C_49_H_58_F_3_N_5_O_3_OsS_2_: C, 54.68; H, 5.43; N, 6.51; S, 5.96. Found: C, 55.02; H, 5.54; N, 6.75; S, 6.28. MS (electrospray, *m*/*z*): C_44_H_50_N_3_OsS [M – 2H – 2CH_3_CN]^+^, 844.3334; found, 844.3371. IR (cm^–1^): ν(Os–H) 2252 (w); ν(NH) 2252 (w); ν(SO_3_) 1248 (s); ν(C= S) 1152 (w); ν(CF_3_) 1030 (s). ^1^H NMR (300 MHz, CD_2_Cl_2_, 298 K): δ 9.16 (s, 1H, N*H*), 7.46–7.35 (4H, Ph), 7.26 (d, ^3^*J*_H–H_ = 7.4, 2H, Ph), 7.20 (s, 2H, *H*-IPr), 7.15–7.06 (3H, Ph), 6.97–6.89 (5H, Ph), 6.72–6.70 (2H, Ph), 2.99 (sept, ^3^*J*_H–H_ = 5.1, 4H, C*H* CH_3_), 2.34 (s, 3H, S=CC*H*_*3*_), 2.31 (s, 3H, N≡CC*H*_*3*_), 1.42 (m, 1H, Os–C*H*_*2*_), 1.42 (d, ^3^*J*_H–H_ = 6.7, 6H, CHC*H*_*3*_), 1.34 (d, ^3^*J*_H–H_ = 6.0, 1H, Os–C*H*_*2*_), 1.31 (s, 3H, N≡CC*H*_*3*_), 1.26 (d, ^3^*J*_H–H_ = 6.7, 6H, CHC*H*_*3*_), 1.20 (d, ^3^*J*_H–H_ = 6.7, 6H, CHC*H*_*3*_), 1.14 (d, ^3^*J*_H–H_ = 6.7, 6H, CHC*H*_*3*_). ^13^C NMR plus HMBC and HSQC (75 MHz, CD_2_Cl_2_, 298 K): δ 198.0 (s, N*C*S), 167.4 (s, N*C*N), 147.6, 146.5, 144.6, 139.0, and 138.9 (all s, C_*ipso*_-CPh + C_*o*_-CPh + C_*ipso*_-Ph), 131.3, 130.7, 128.0, 127.6, and 127.1 (all s, CPh + Ph), 126.6 (s, *C*-IPr), 125.7, 124.2, 124.1, and 123.8 (all s, CPh + Ph + *C*-IPr), 121.0 (s, N≡*C*CH_3_), 120.9 (s, N≡*C*CH_3_), 94.5 (s, N*C*Ph), 79.4 (s, Os*C* Ph), 29.8 (s, Os-*C*H_2_), 29.3 and 29.2 (both s, *C*HCH_3_), 26.9, 26.5, 23.8, 23.3, and 23.2 (all s, CH*C*H_3_), 6.0 (s, N≡C*C*H_3_), 2.7 (s, N≡C*C*H_3_).

### Preparation of OsH_2_(C≡CPh){κ^2^-*C,S*-[C(Ph)NC(CH_3_)S]}(IPr)(P^i^Pr_3_) (**7**)

Phenylacetylene (13.5 μL, 0.12 mmol) was added to a dark purple solution of **5** (100 mg, 0.11 mmol) in toluene (5 mL) at room temperature. The resulting dark brown mixture was stirred for 5 min. After that time, the solution was concentrated under vacuum almost to dryness. Then, cold pentane (0 °C) was added, giving a brown solid which was washed again with cold pentane (3 × 5 mL). Yield: 49 mg (44%). Anal. Calcd for C_53_H_72_N_3_OsPS: C, 63.38; H, 7.23; N, 4.18; S, 3.19. Found: C, 63.67; H, 7.45; N, 4.27; S, 3.51. MS (electrospray, *m*/*z*): C_53_H_71_N_3_OsPS [M – H]^+^, 1004.4715; found, 1004.4690. IR (cm^–1^): ν(C≡C) 2099 (w); ν(C=S) 1384 (s). ^1^H NMR (400 MHz, Tol-*d*_8_, 298 K) δ 8.47 (d, ^3^*J*_H–H_ = 8.0, 2H, *o*-Ph), 7.29–7.20 (3H, Ph), 7.16–7.14 (2H, Ph), 7.08–7.06 (2H, Ph), 7.02 (br, 2H, Ph), 6.30 (s, 2H, *H*-IPr), 3.05 (sept, ^3^*J*_H–H_ = 6.7, 2H, C*H*CH_3_), 2.92 (s, 3H, S=CC*H*_*3*_), 2.82 (sept, ^3^*J*_H–H_ = 6.7, 2H, C*H*CH_3_), 1.75 (d, ^3^*J*_H–H_ = 6.8, 6H, CHC*H*_*3*_), 1.44 (sept, ^3^*J*_H–H_ = 7.6, 3H, PC*H*CH_3_), 1.30 (d, ^3^*J*_H–H_ = 6.8, 6H, CHC*H*_*3*_), 1.08 (d, ^3^*J*_H–H_ = 6.8, 6H, CHC*H*_*3*_), 0.94 (d, ^3^*J*_H–H_ = 6.8, 6H, CHC*H*_*3*_), 0.62 (dd, ^2^*J*_H–P_ = 12.3, ^3^*J*_H–H_ = 6.9, 9H, PCHC*H*_*3*_), 0.60 (dd, ^2^*J*_H–P_ = 12.3, ^3^*J*_H–H_ = 6.9, 9H, PCHC*H*_*3*_), −7.89 (d, ^2^*J*_H–P_ = 15.7, 2H, Os-*H*_*2*_). T_1_ (min) (ms, Os–H_2_, 300 MHz, toluene-*d*_8_, 243 K): 97 ± 4 (−7.93 ppm). ^31^P {^1^H} NMR (121 MHz, Tol-*d*_8_, 298 K): δ 29.3 (s). ^13^C NMR plus HMBC and HSQC (101 MHz, Tol-*d*_8_, 298 K): δ 272.4 (s, Os*C*), 204.1 (s, N*C*S), 168.5 (d, ^2^*J*_C–P_ = 57.8, N*C*N), 161.7 (s, *C*_*ipso*_-Ph), 146.8, 146.21, and 138.37 (all s, *C*_*ipso*_–CPh + *C*_*o*_-CPh), 131.0, 129.3, 126.4, 123.6, 123.3, and 123.1 (all s, Ph + CPh + *C*-IPr), 29.1 and 28.6 (both s, *C*HCH_3_), 28.1 (d, ^3^*J*_C–P_ = 25.0, P*C*HCH_3_), 25.8 and 25.5 (both s, CH*C*H_3_), 22.7 and 21.50 (all s, CH*C*H_3_), 19.3 and 19.2 (both s, PCH*C*H_3_) The resonances corresponding to C_α_ and C_β_ of the alkynyl ligand are overlapped with those of toluene-*d*_8_.

### Preparation of Os(C≡CPh){κ^2^-*C,S*-[C(Ph)NC(CH_3_)S]}(IPr)(P^i^Pr_3_) (**8**)

Complex **7** (100 mg, 0.10 mmol) was stirred in acetonitrile (5 mL) for 1 h. The resulting purple solution was evaporated under vacuum almost to dryness. Then, pentane (3 mL) was added to afford a purple solid, which was washed with more pentane (3 × 3 mL). Yield: 58 mg (53%). Crystals suitable for X-ray analysis were formed in acetonitrile at 4 °C in a drybox. Anal. Calcd for C_53_H_70_N_3_OsPS: C, 63.50; H, 7.04; N, 4.19; S, 3.20. Found: C, 63.17; H, 7.26; N, 4.34; S, 3.54. MS (electrospray, *m*/*z*): C_53_H_71_N_3_OsPS [M + H]^+^, 1004.4715; found, 1004.4733. IR (cm^–1^): ν(C≡C) 2036 (w); ν(C=S) 1593 (s). ^1^H NMR (400 MHz, Tol-*d*_8_, 273 K) δ 8.78 (d, ^3^*J*_H–H_ = 7.5, 2H, *o*-Ph), 7.51 (d, ^3^*J*_H–H_ = 7.2, 2H, *o*-Ph), 7.27–6.95 (10H, Ph), 6.79 (t, ^3^*J*_H–H_ = 7.7, 1H, Ph), 6.61 (d, ^3^*J*_H–H_ = 7.7, 1H, Ph), 6.43 (d, ^3^*J*_H–H_ = 1.6, 1H, *H*-IPr), 6.37 (d, ^3^*J*_H–H_ = 1.6, 1H, *H*-IPr), 6.43 (s, 1H, *H*-IPr), 6.37 (s, 1H, *H*-IPr), 4.24 (sept, ^3^*J*_H–H_ = 6.7, 1H, C*H*CH_3_), 3.54 (sept, ^3^*J*_H–H_ = 6.7, 1H, C*H*CH_3_), 3.43 (s, 3H, S=CC*H*_*3*_), 3.34 (sept, ^3^*J*_H–H_ = 6.7, 1H, C*H*CH_3_), 2.41 (sept, ^3^*J*_H–H_ = 7.4, 3H, PC*H*CH_3_), 2.32 (sept, ^3^*J*_H–H_ = 6.7, 1H, C*H*CH_3_), 1.79 (d, ^3^*J*_H–H_ = 6.6, 3H, CHC*H*_*3*_), 1.69 (d, ^3^*J*_H–H_ = 6.6, 3H, CHC*H*_*3*_), 1.53 (d, ^3^*J*_H–H_ = 6.6, 3H, CHC*H*_*3*_), 1.31 (d, ^3^*J*_H–H_ = 6.6, 3H, CHC*H*_*3*_), 1.08 (d, ^3^*J*_H–H_ = 6.6, 6H, CHC*H*_*3*_), 1.04 (d, ^3^*J*_H–H_ = 6.6, 3H, CHC*H*_*3*_), 0.81 (d, ^3^*J*_H–H_ = 6.6, 3H, CHC*H*_*3*_), 0.74 (dd, ^2^*J*_H–P_ = 13.0, ^3^*J*_H–H_ = 5.8, 9H, PCHC*H*_*3*_), 0.65 (dd, ^2^*J*_H–P_ = 13.0, ^3^*J*_H–H_ = 5.8, 9H, PCHC*H*_*3*_). ^31^P {^1^H} NMR (121 MHz, Tol-*d*_8_, 298 K): δ 21.4 (s). ^13^C NMR plus HMBC and HSQC (101 MHz, Tol-*d*_8_, 273 K): δ 245.1 (d, ^2^*J*_C–P_ = 4.5, Os*C*), 208.7 (s, N*C*S), 185.2 (d, ^2^*J*_C–P_ = 63.0, N*C*N), 152.1 (s, *C*_*ipso*_-Ph), 147.3, 146.8, 145.8, 144.9, 136.9, and 135.8 (all s, *C*_*ipso*_-CPh + *C*_*o*_-CPh), 130.8 (s, *C*_*ipso*_-C≡C-*Ph*), 130.6 (d, ^2^*J*_C–P_ = 9.4, Os-*C*≡), 130.8, 130.5, 129.9, 127.9, 127.6, 126.7, 126.0, 124.9, 124.5, 124.3, 123.8, and 122.8 (all s, Ph + CPh + *C*-IPr), 115.7 (s, ≡*C*-Ph), 29.4, 28.8, 28.7, and 27.9 (all s, *C*HCH_3_), 27.2, 26.9, 26.4, 26.3, and 26.0 (all s, CH*C*H_3_), 23.7 (d, ^3^*J*_C–P_ = 24.8, P*C*HCH_3_), 23.5, 22.7, and 22.2 (all s, CH*C*H_3_), 20.4 and 20.1 (both s, PCH*C*H_3_).

## Supporting Information Available

General information, structural analysis of complexes **2**–**6** and **8**, computational data, and NMR spectra (PDF)
Cartesian coordinates of computed complexes (XYZ)

## Supplementary Material

om2c00631_si_001.pdf

om2c00631_si_002.xyz
