# Supplementary material for: Osmathiazole Ring: Extrapolation of an Aromatic Purely Organic System to Organometallic Chemistry
Source: Organometallics. 2023 Feb 9;42(4):327–38. doi: 10.1021/acs.organomet.2c00631 (PMC11005464; doi:10.1021/acs.organomet.2c00631)
Supplement: Supplementary file 1 — om2c00631_si_001.pdf [file om2c00631_si_001.pdf]

SUPPORTING INFORMATION

Osmathiazole Ring: The Extrapolation of an Aromatic Purely Organic System to Organometallic Chemistry

María L. Buil, Miguel A. Esteruelas,* Enrique Oñate, and Nieves R. Picazo.

Departamento de Química Inorgánica, Instituto de Síntesis Química y Catálisis Homogénea (ISQCH), Centro de Innovación en Química Avanzada (ORFEO-CINQA), Universidad de Zaragoza-CSIC, 50009 Zaragoza, Spain

* email: maester@unizar.es

Contents:

- Experimental Details. **S2**

- Structural Analysis of Complexes 2, 3, 4, 5, 6 and 8. **S2**

- NMR Spectra. **S5**

- Computational Details. **S17**

- Energies of Optimized Structures. **S17**

- AICD plots **S19**

- NICS and NICS$_{zz}$ scans **S20**

- NBO π-bond orbitals. **S22**

- X-ray Bond Lengths **S24**

- Bond Wiberg **S24**

- NBO Charges **S24**

- References. **S25**

- **Experimental details.**

General information: All reactions were carried out with exclusion of air using Schlenk-tube techniques or in a drybox. Diethyl ether, pentane, acetonitrile, dichloromethane and toluene were obtained oxygen- and water-free from an MBraun solvent purification apparatus, while tetrahydrofuran was dried and distilled under argon prior to use. ^{1}H, ^{13}C{^{1}H}, and ^{31}P{^{1}H} NMR spectra were recorded on Bruker 300 ARX, Bruker Advance 300 MHz or Bruker Advance 400MHz. Coupling constants *J* are given in hertz. Attenuated total reflection infrared spectra (ATR-IR) of solid samples were run on a PerkinElmer Spectrum 100 FT-IR spectrometer. Elemental analyses were carried out in a PerkinElmer 2400 CHNS/O analyzer. High-resolution electrospray mass spectra were acquired using a MicroTOF-Q hybrid quadrupole time-of-flight spectrometer (Bruker Daltonics, Bremen, Germany).

- **Structural Analysis of Complexes 2, 3, 4, 5, 6, and 8.**

X-ray data were collected on a APEX D8 Venture Bruker diffractometer (Mo radiation, λ = 0.71073 Å). The crystals were cooled with a nitrogen flow with a Oxford Cryosystems system. Data were corrected for absorption by using a multiscan method applied with the SADABS program.[1] The structures were solved by Patterson or direct methods and refined by full-matrix least squares on F^2 with SHELXL2019,[2] including isotropic and subsequently anisotropic displacement parameters. The hydrogen atoms were observed in the last Fourier Maps or calculated, and refined freely or using a restricted riding model. The disordered molecules were refined with restrained geometries and isotropic displacement parameters.

Crystal data for **2**: $C_{45}H_{67}N_3OsPS$, CF_3O_3S, $2(C_4H_8O)$, M_W 1196.52, red, irregular block, (0.162 x 0.092 x 0.041 mm^3), orthorhombic, space group $P2_12_12_1$, *a*: 11.1260(4)

Å, b: 20.4174(6) Å, c: 24.5109(7) Å, V = 5568.0(3) Å^3, Z = 4, Z' = 1, D_{calc}: 1.493 g cm^{-3}, F(000): 2472, T = 100(2) K, μ 2.451 mm^{-1}. 128706 measured reflections (2θ: 3-57°, ω and φ scans 0.5°), 13828 unique (R_{int} = 0.0358); min./max. transm. Factors 0.716/0.862. Final agreement factors were R^1 = 0.0224 (13725 observed reflections, I > 2σ(I)) and wR^2 = 0.0625; Flack parameter 0.426(5); data/restraints/parameters 13828/26/615; GoF = 0.998. Largest peak and hole 1.520 (close to Os atoms) and -0.740 e/ Å^3.

Crystal data for **3**: $C_{47}H_{70}N_4OsPS$, CF_3O_3S, M_W 1093.37, red, irregular block (0.200 x 0.100 x 0.050 mm^3), monoclinic, space group Cc, a: 12.5209(4) Å, b: 22.7312(8) Å, c: 17.1800(5) Å, β: 95.8759(10)°, V = 4864.0(3) Å^3, Z = 4, Z' = 1, D_{calc}: 1.493 g cm^{-3}, F(000): 2240, T = 100(2) K, μ 2.796 mm^{-1}. 75648 measured reflections (2θ: 3-57°, ω and φ scans 0.5°), 11403 unique (R_{int} = 0.0244); min./max. transm. Factors 0.728/0.862. Final agreement factors were R^1 = 0.0123 (11379 observed reflections, I > 2σ(I)) and wR^2 = 0.0282; Flack parameter -0.011(2); data/restraints/parameters 11403/2/582; GoF = 1.025. Largest peak and hole 0.518 (close to Ir atoms) and -0.875 e/ Å^3.

Crystal data for **4**: $C_{42}H_{55}N_6OsS$, CF_3O_3S, M_W 1015.25, yellow, irregular block, (0.059 x 0.036 x 0.032 mm^3), monoclinic, space group $P2_1$, a: 11.4742(3) Å, b: 23.5013(7) Å, c: 17.4998(5) Å, β: 99.5879(11)°, V = 4653.1(2) Å^3, Z = 4, Z' = 2, D_{calc}: 1.449 g cm^{-3}, F(000): 2056, T = 100(2) K, μ 2.885 mm^{-1}. 163845 measured reflections (2θ: 3-51°, ω and φ scans 0.5°), 23087 unique (R_{int} = 0.0496); min./max. transm. Factors 0.801/0.862. Final agreement factors were R^1 = 0.0470 (22401 observed reflections, I > 2σ(I)) and wR^2 = 0.1192; data/restraints/parameters 23087/19/ 1080; GoF = 1.209. Largest peak and hole 6.441 (close to Os atoms) and -3.140 e/ Å^3.

Crystal data for **5**: $C_{45}H_{66}N_3OsPS$, M_W 902.23, violet, irregular block, (0.100 x 0.094 x 0.052 mm^3), monoclinic, space group $P2_1/n$, a: 11.9923(3) Å, b: 17.9565(5) Å, c: 20.3919(6) Å, β: 103.4751(9)°, V = v Å^3, Z = 4, Z' = 1, D_{calc}: 1.403 g cm^{-3}, F(000):

1856, T = 100(2) K, μ 3.106 mm^{-1}. 284271 measured reflections (2θ: 3-57°, ω and φ scans 0.5°), 10597 unique (R_{int} = 0.0381); min./max. transm. Factors 0.812/0.862. Final agreement factors were R^1 = 0.0201 (10177 observed reflections, I > 2σ(I)) and wR^2 = 0.0464; data/restraints/parameters 10597/1/477; GoF = 1.058. Largest peak and hole 1.866 (close to Os atoms) and -0.898 e/ $Å^3$.

Crystal data for **6**: $C_{48}H_{58}N_5OsS$, CF_3O_3S, 1.5(CH_2Cl_2), M_W 1203.71, orange, irregular block (0.177 x 0.160 x 0.077 mm^3), triclinic, space group P-1, *a*: 11.0827(5) Å, *b*: 11.1679(5) Å, *c*: 22.6601(9) Å, *α*: 75.883(2)°, *β*: 82.384(2)°, *γ*: 89.223(2)°, *V* = 2695.4(2) $Å^3$, *Z* = 2, *Z'* = 1, D_{calc}: 1.483 g cm^{-3}, F(000): 1218, T = 100(2) K, μ 2.646 mm^{-1}. 194049 measured reflections (2θ: 3-57°, ω and φ scans 0.5°), 16466 unique (R_{int} = 0.0312); min./max. transm. Factors 0.701/0.862. Final agreement factors were R^1 = 0.0465 (15861 observed reflections, I > 2σ(I)) and wR^2 = 0.1293; data/restraints/parameters 16466/47/ 597; GoF = 1.068. Largest peak and hole 3.817 (close to S atoms) and -2.779 e/ $Å^3$.

Crystal data for **8**: $C_{53}H_{70}N_3OsPS$, 2(C_2H_3N), M_W 1084.45, violet, irregular block (0.534 x 0.106 x 0.062 mm^3), monoclinic, space group $P2_1/c$, *a*: 17.5789(5) Å, *b*: 13.3149(4) Å, *c*: 22.5432(7) Å, *β*: 94.0965(10)°, *V* = 5263.0(3) $Å^3$, *Z* = 4, *Z'* = 1, D_{calc}: 1.369 g cm^{-3}, F(000): 2240, T = 100(2) K, μ 2.534 mm^{-1}. 146785 measured reflections (2θ: 3-57°, ω and φ scans 0.5°), 13087 unique (R_{int} = 0.0351); min./max. transm. Factors 0.565/0.862. Final agreement factors were R^1 = 0.0177 (12320 observed reflections, I > 2σ(I)) and wR^2 = 0.0406; data/restraints/parameters 13087/0/603; GoF = 1.038. Largest peak and hole 1.887 (close to Os atoms) and -0.621 e/ $Å^3$.

- **NMR Spectra.**

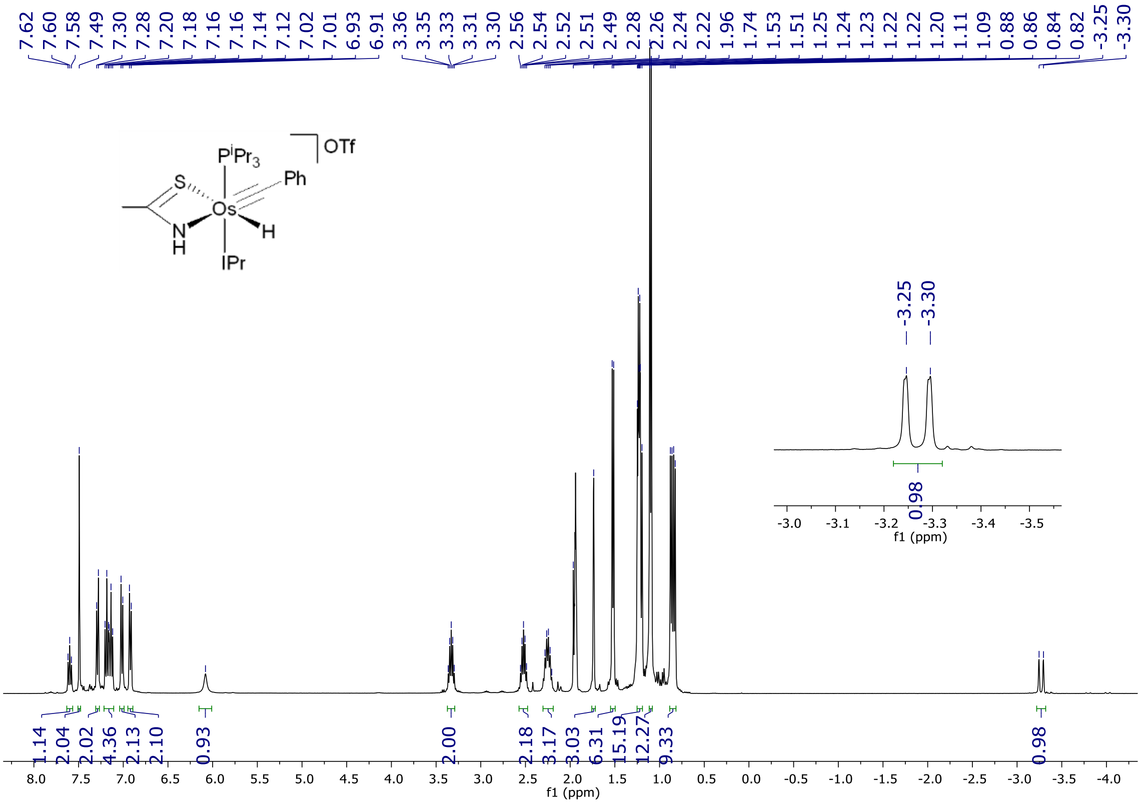

Figure S1. ^{1}H NMR spectrum (400 MHz, CD_3CN, 298 K) of compound **2**.

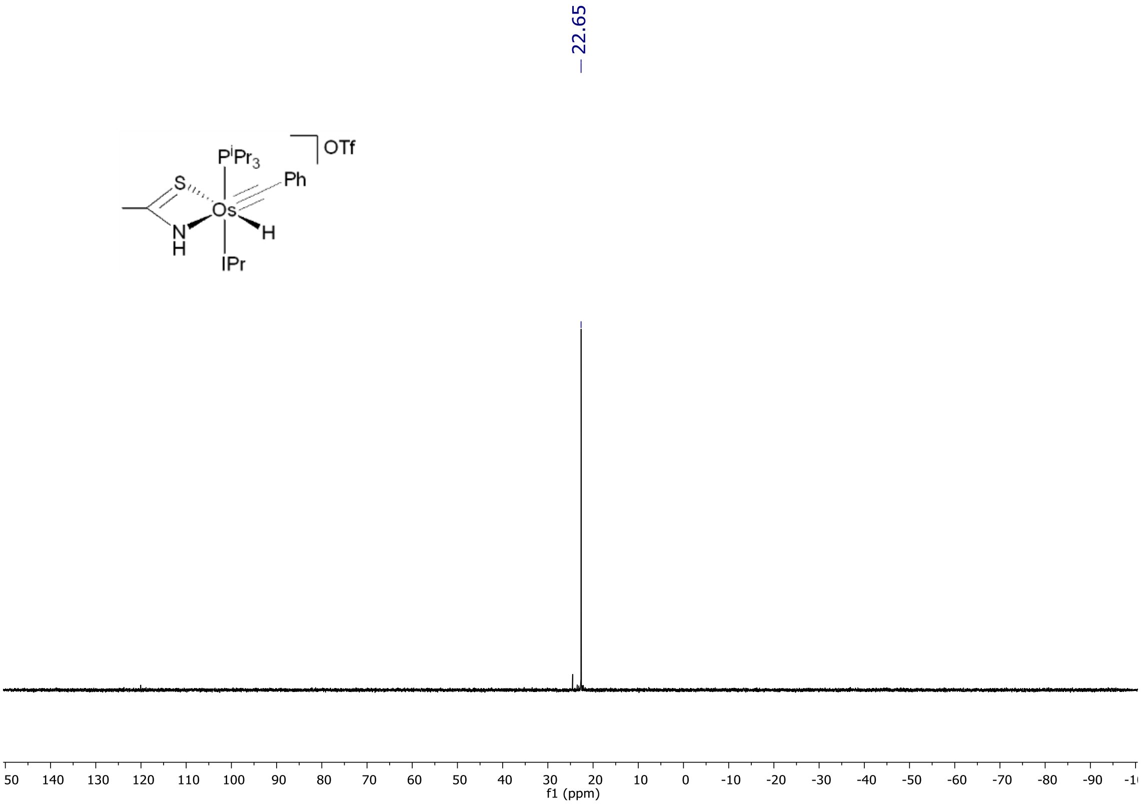

Figure S2. ^{31}P{^{1}H} spectrum (121 MHz, CD_3CN, 298 K) of compound **2**.

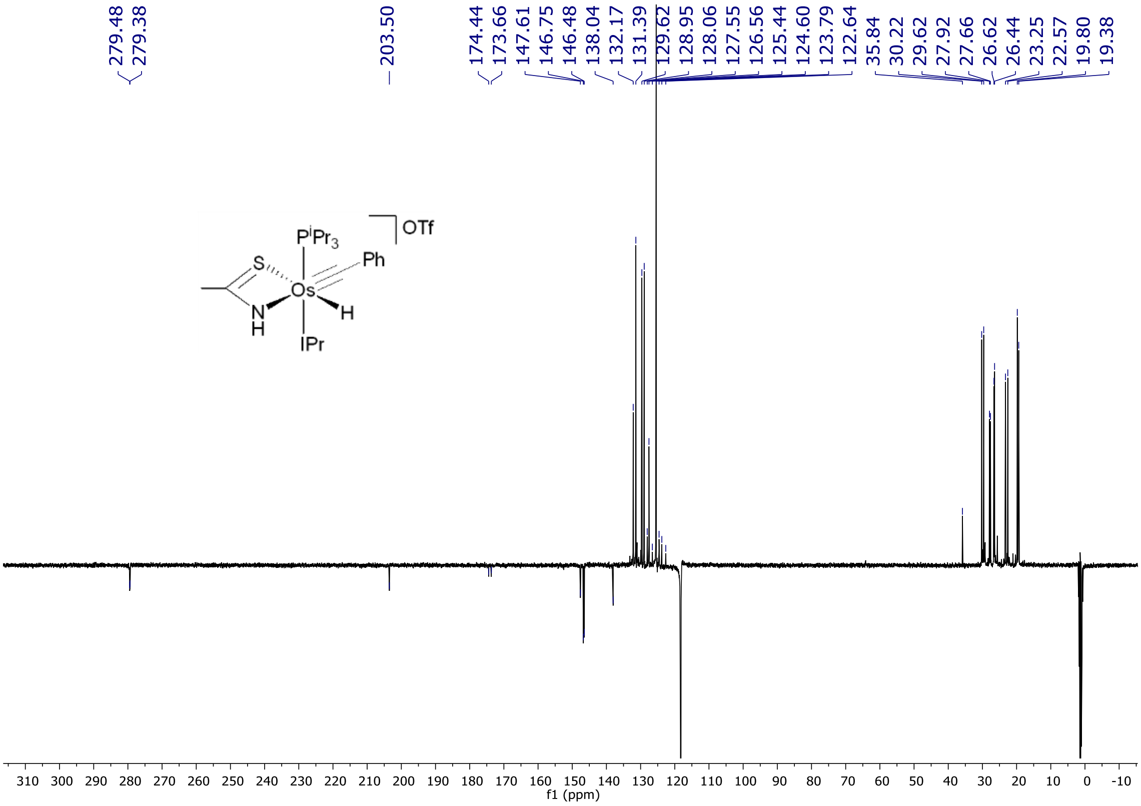

Figure S3. $^{13}C\{^1H\}$ spectrum (101 MHz, CD_3CN, 298 K) of compound **2**.

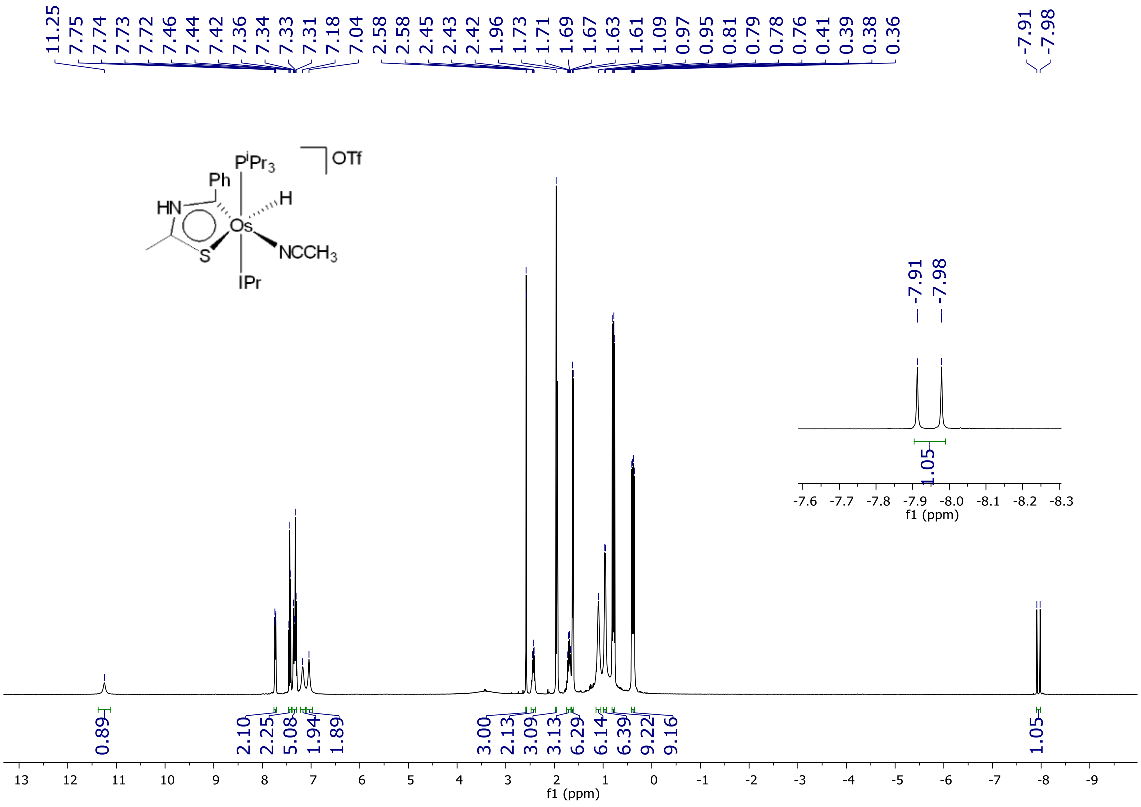

Figure S4. ^1H NMR spectrum (400 MHz, CD_3CN, 298 K) of compound **3**.

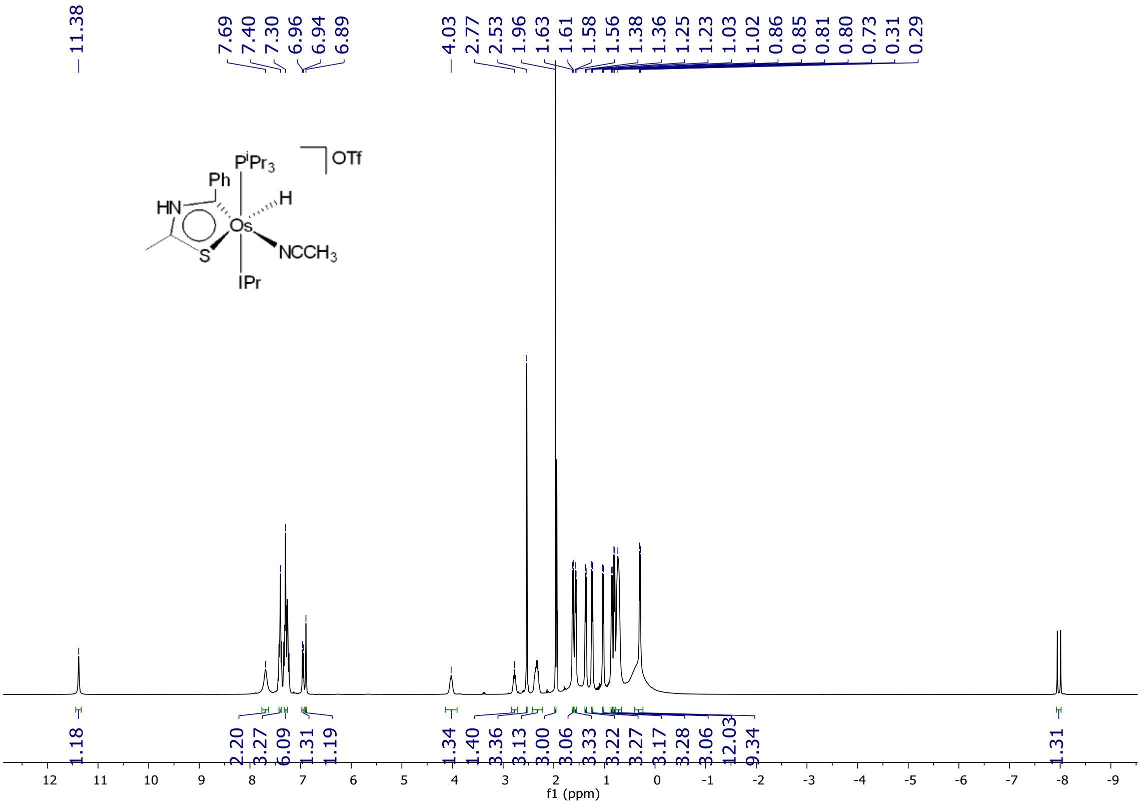

Figure S5. ^{1}H NMR spectrum (400 MHz, CD_3CN, 263 K) of compound **3**.

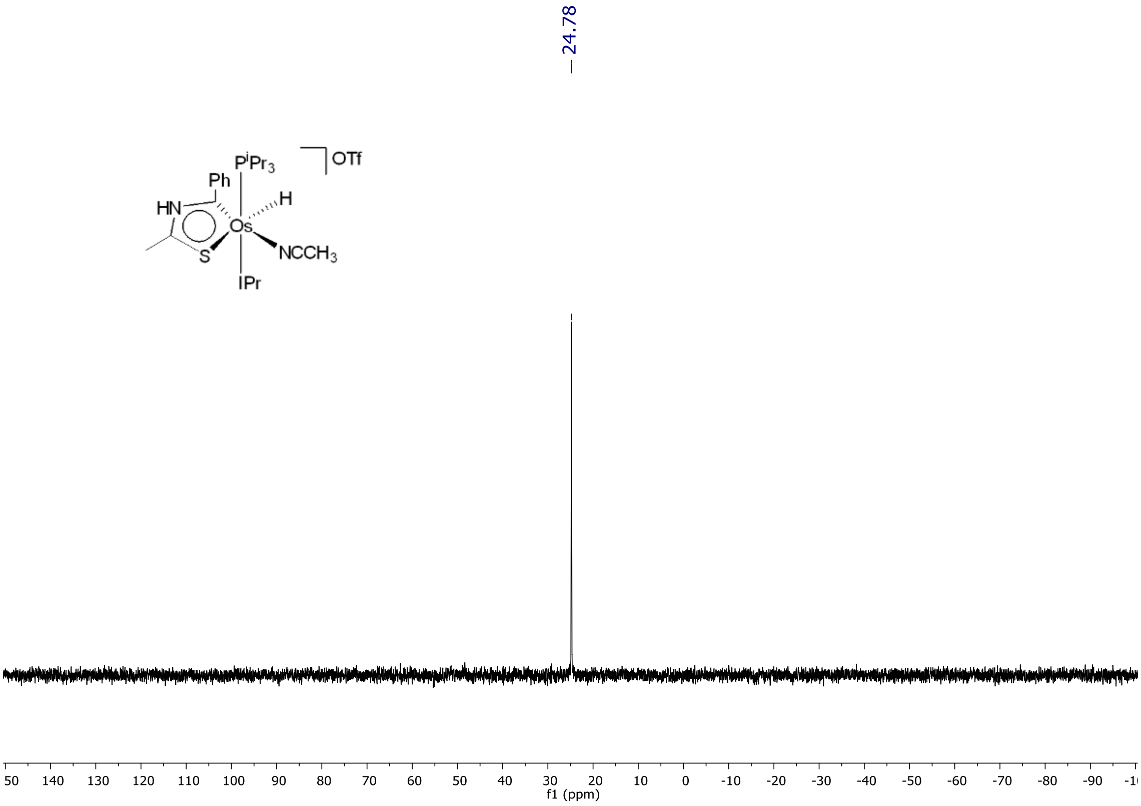

Figure S6. ^{31}P{^{1}H} spectrum (162 MHz, CD_3CN, 298 K) of compound **3**.

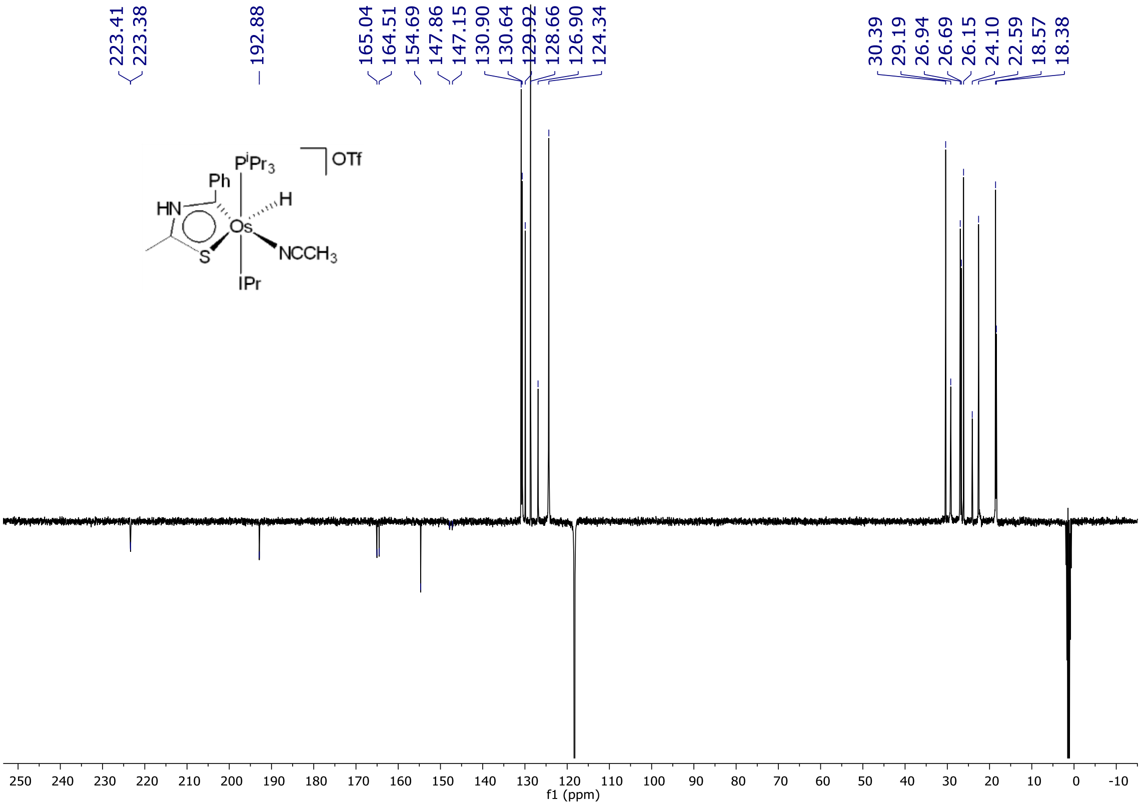

Figure S7. $^{13}C\{^1H\}$ spectrum (101 MHz, CD_3CN, 298 K) of compound **3**.

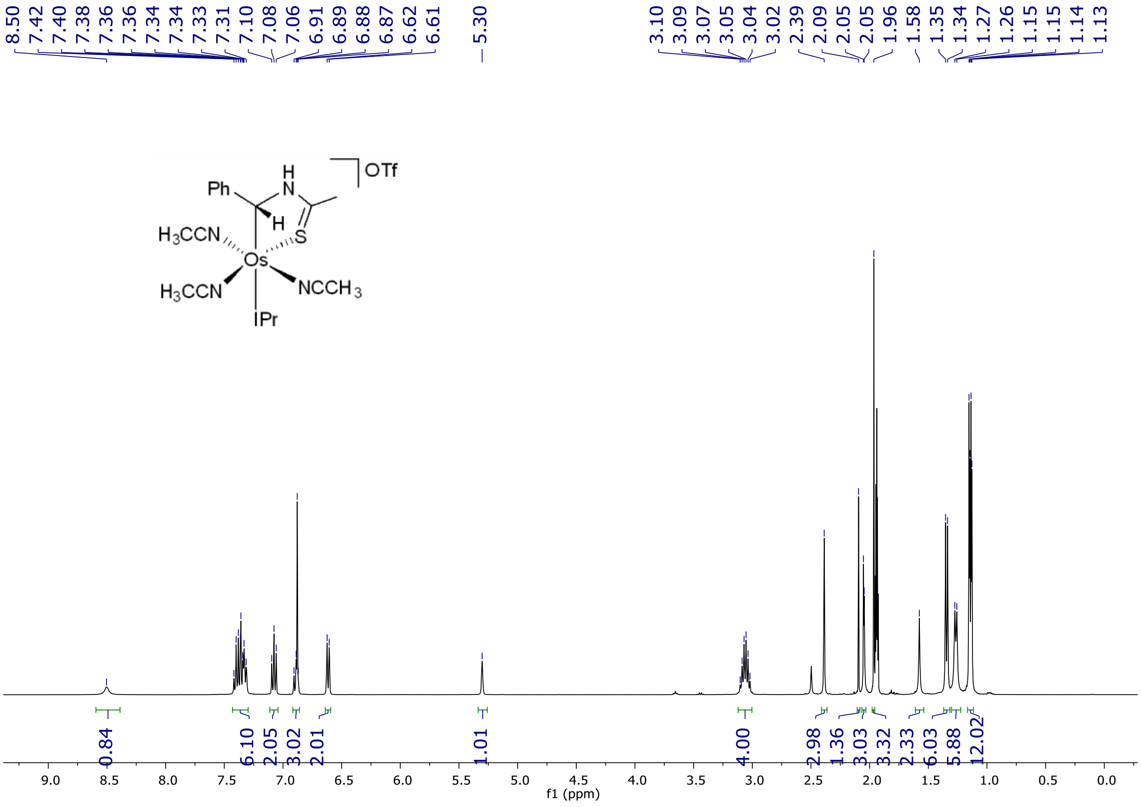

Figure S8. ^1H NMR spectrum (400 MHz, CD_3CN, 330 K) of compound **4**.

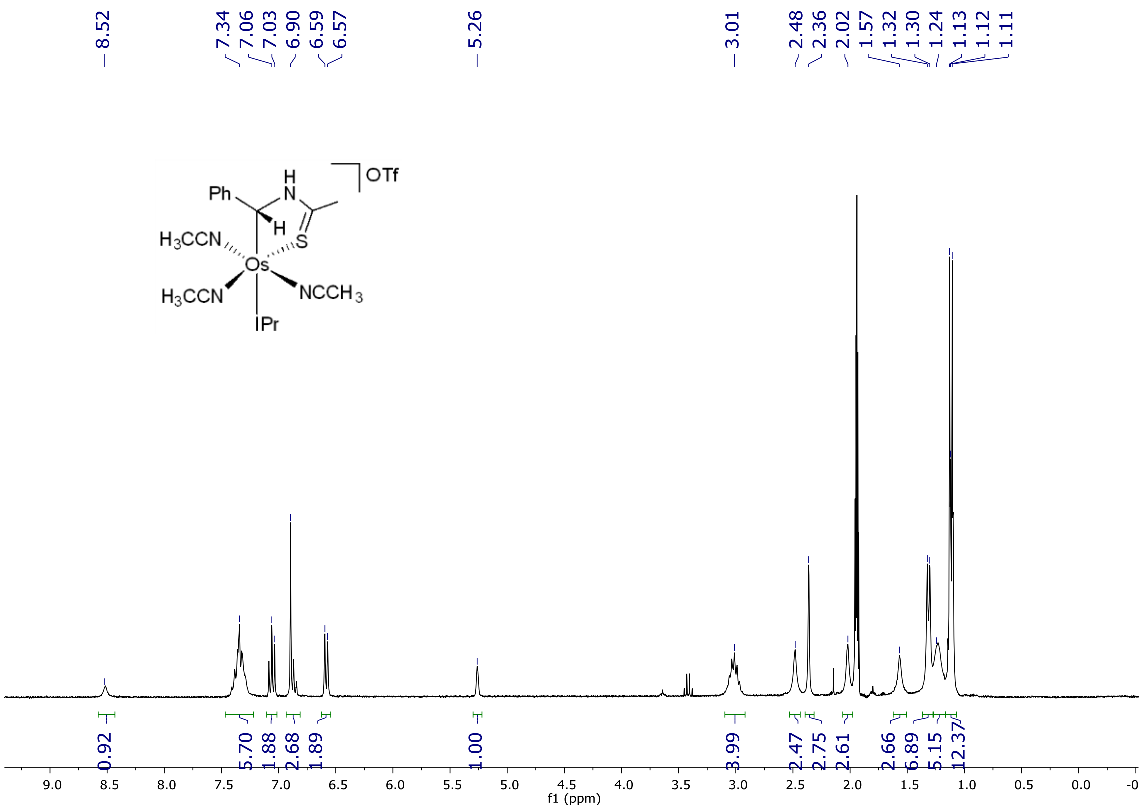

Figure S9. ^{1}H NMR spectrum (400 MHz, CD_3CN, 298 K) of compound **4**.

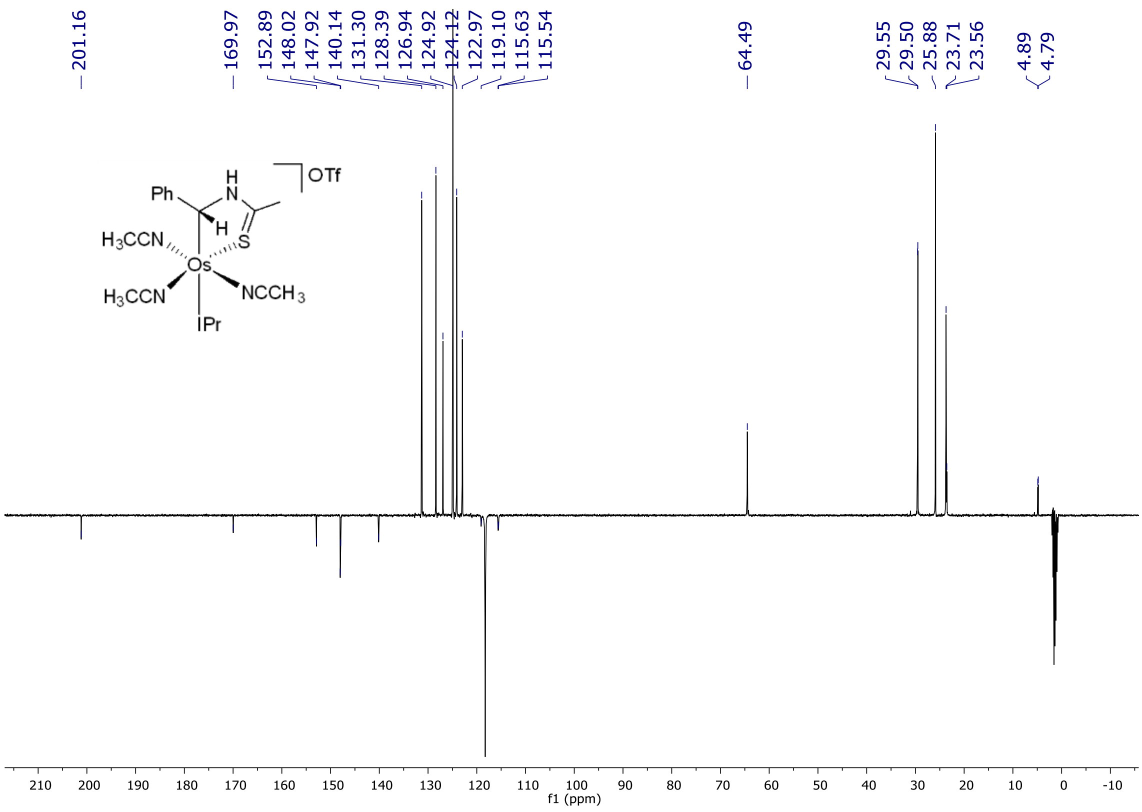

Figure S10. $^{13}C\{^1H\}$ spectrum (101 MHz, CD_3CN, 330 K) of compound **4**.

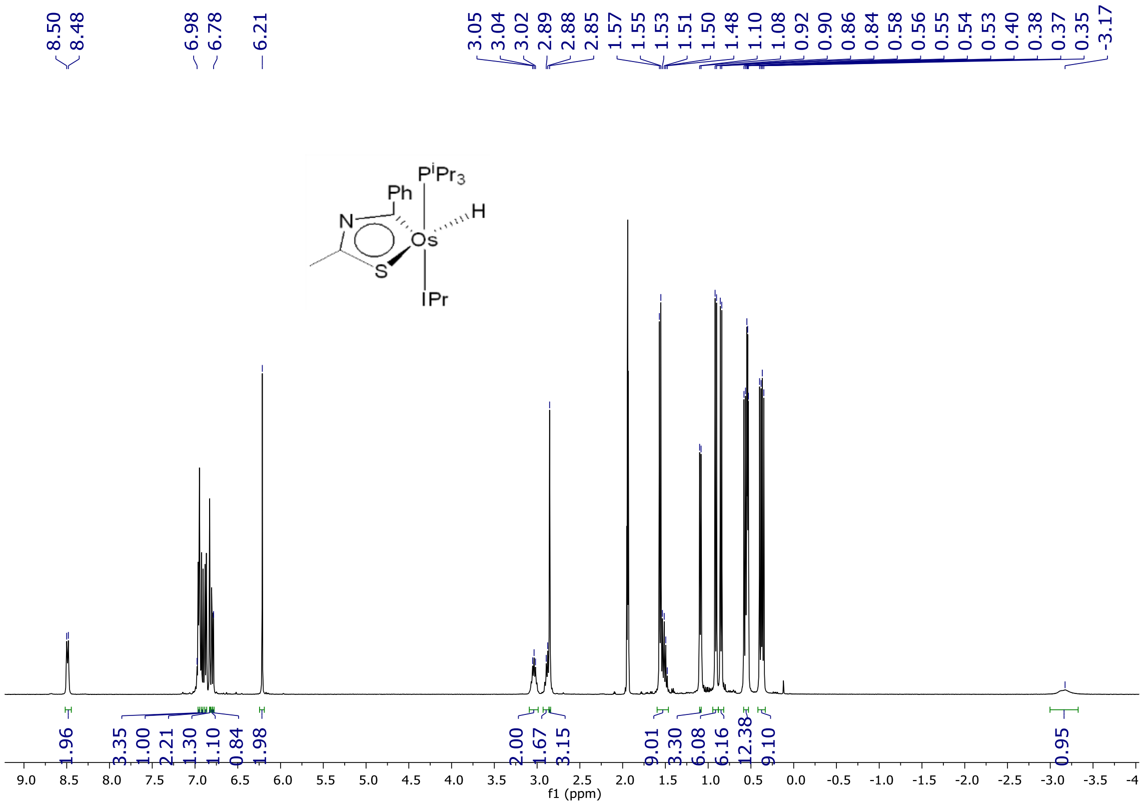

Figure S11. ^{1}H NMR spectrum (400 MHz, Tol-d_8, 298 K) of compound **5**.

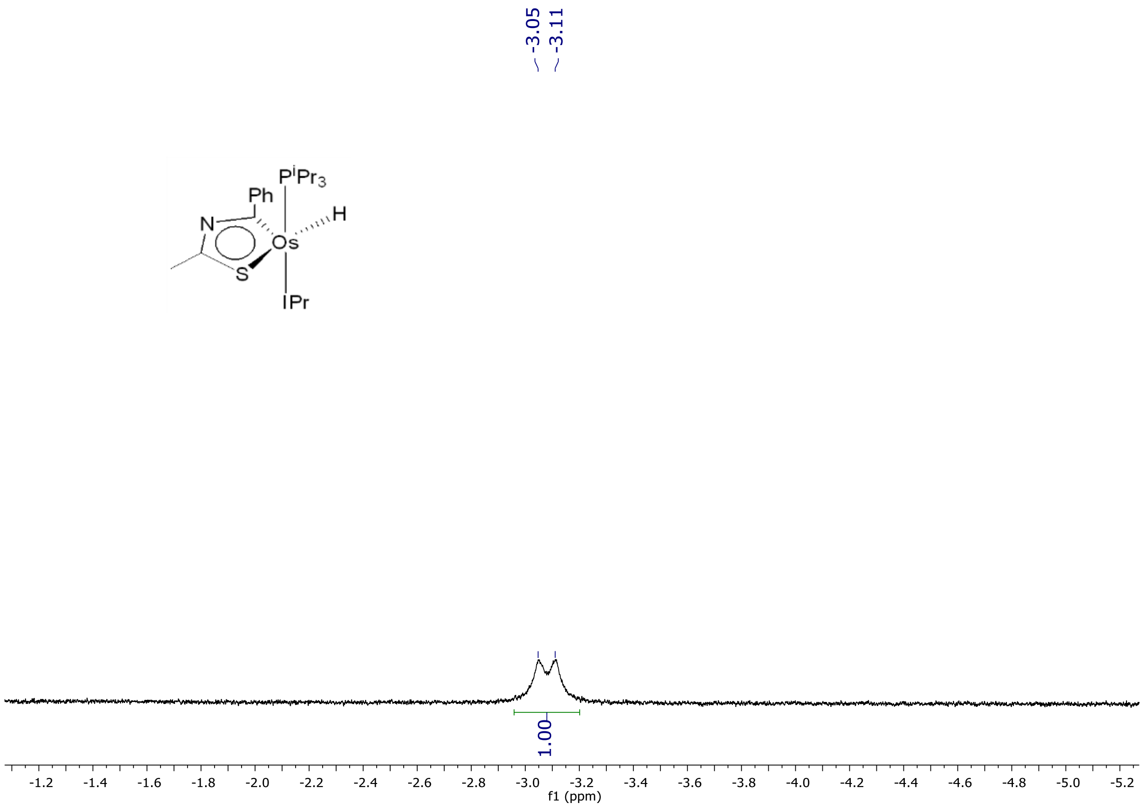

Figure S12. High field region of the ^{1}H NMR spectrum (400 MHz, Tol-d_8, 213 K) of compound **5**.

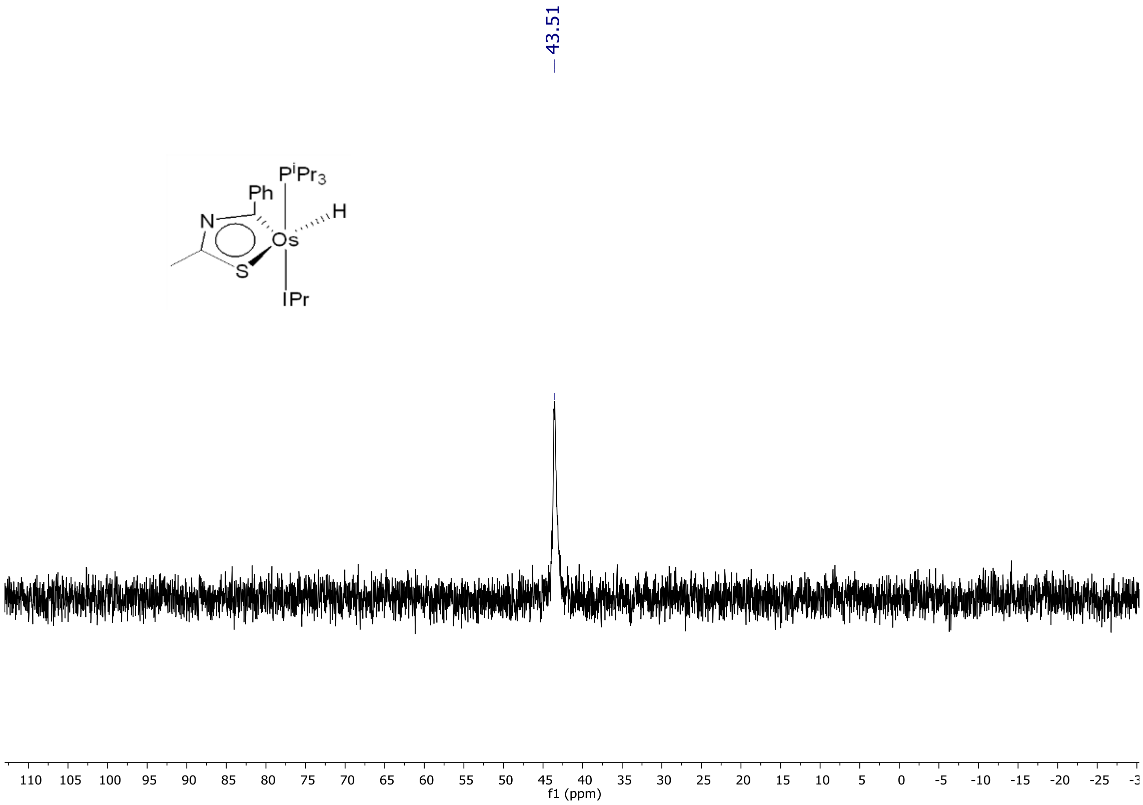

Figure S13. ^{31}P{^{1}H} spectrum (162 MHz, Tol-d_8, 298 K) of compound **5**.

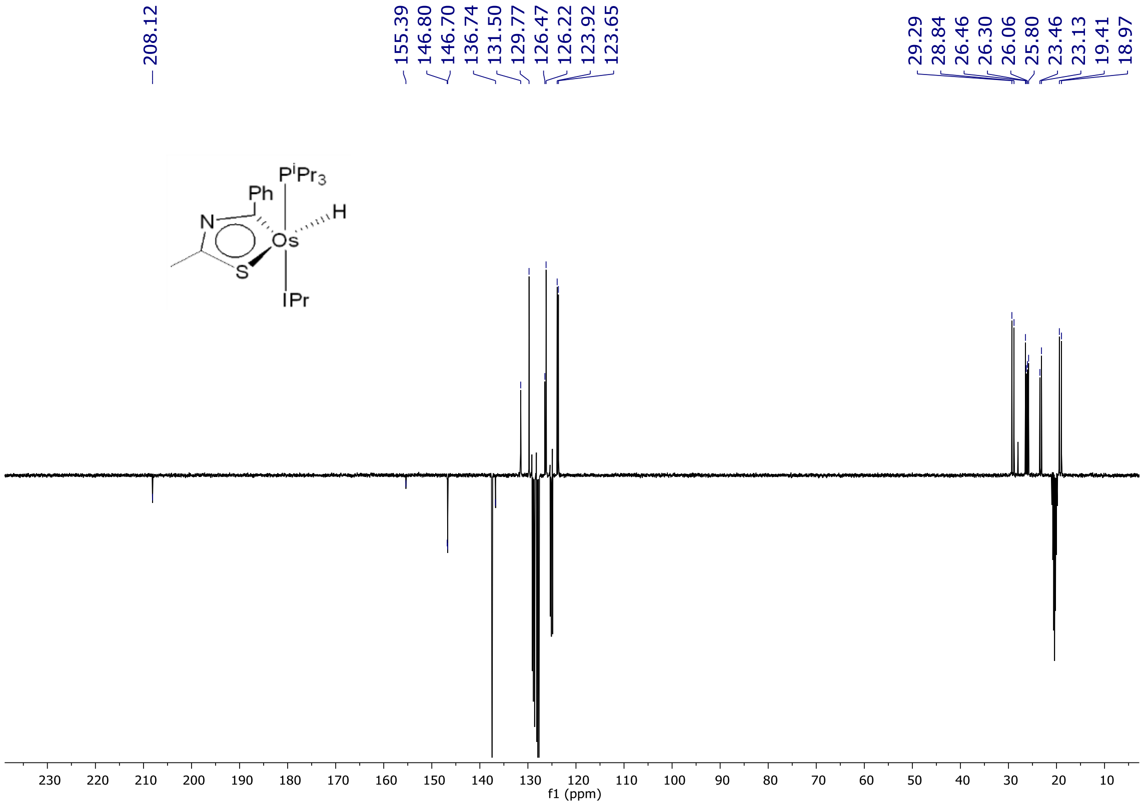

Figure S14. ^{13}C{^{1}H} spectrum (101 MHz, Tol-d_8, 298 K) of compound **5**.

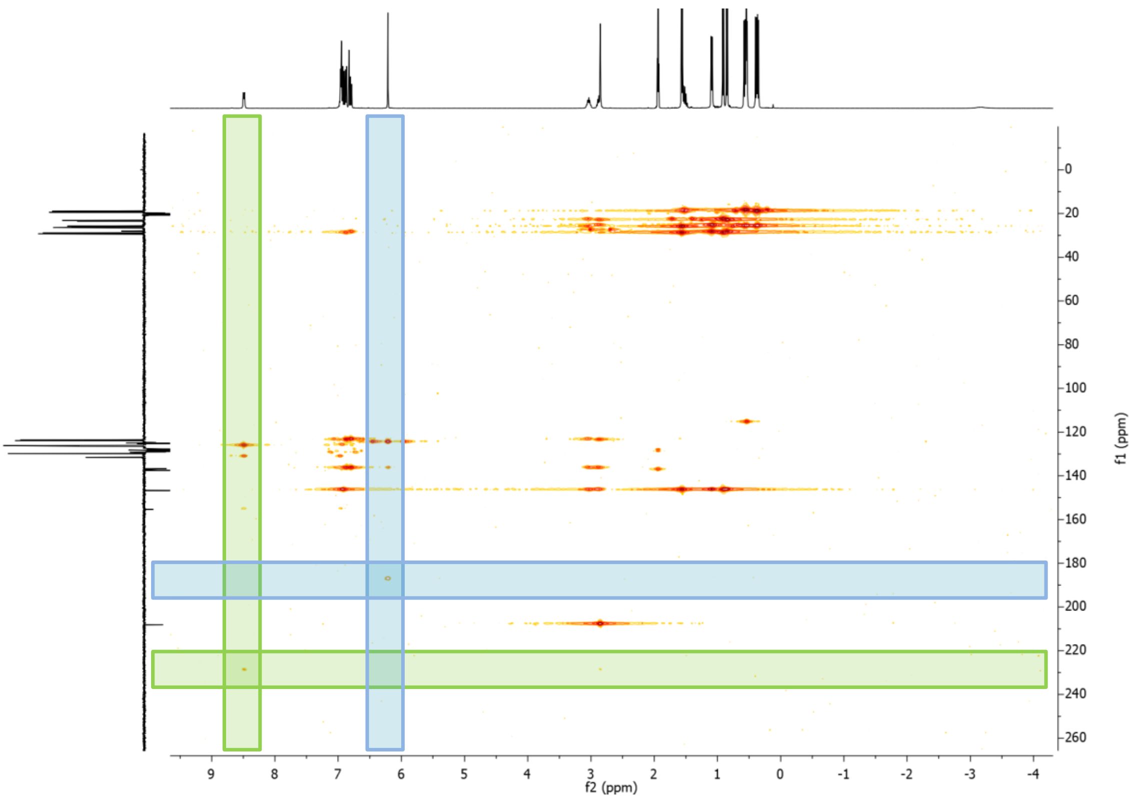

Figure S15. HMBC ^{1}H-$^{13}C\{^{1}H\}$ spectrum (101 MHz, Tol-d_8, 298 K) of compound **5**.

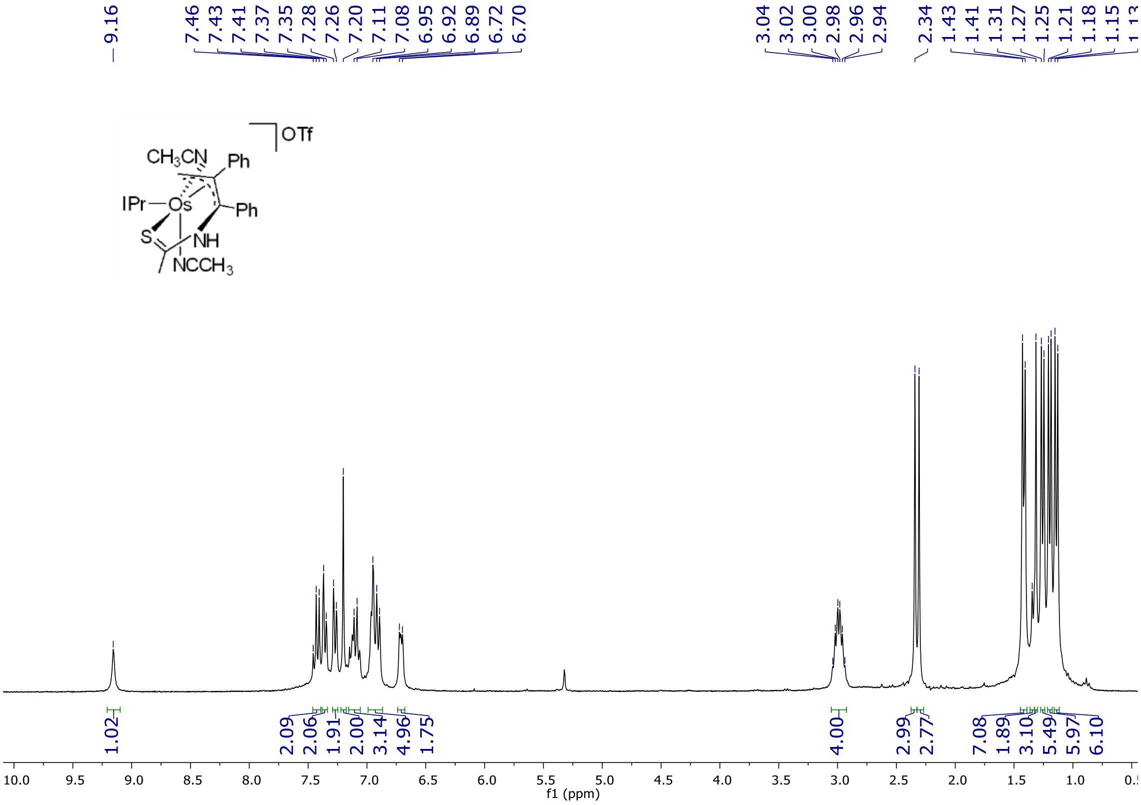

Figure S16. ^{1}H NMR spectrum (300 MHz, CD_2Cl_2, 298 K) of compound **6**.

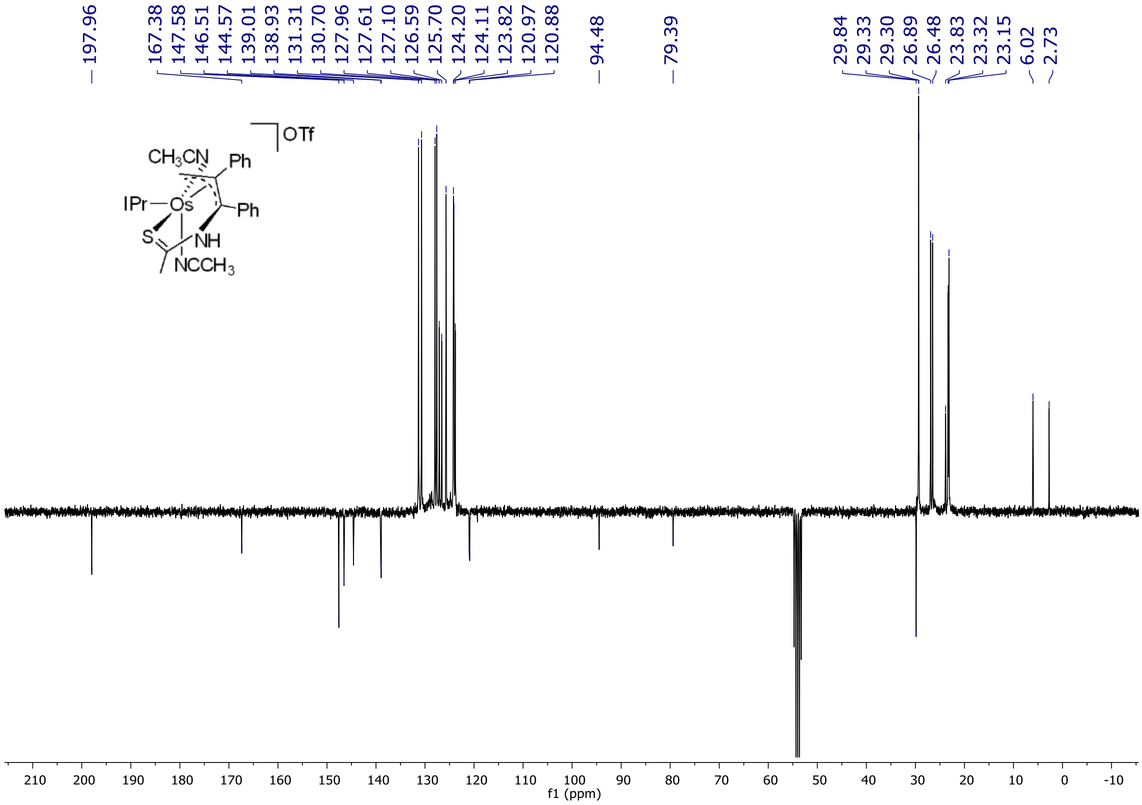

Figure S17. $^{13}C\{^{1}H\}$ spectrum (75 MHz, CD_2Cl_2, 298 K) of compound **6**.

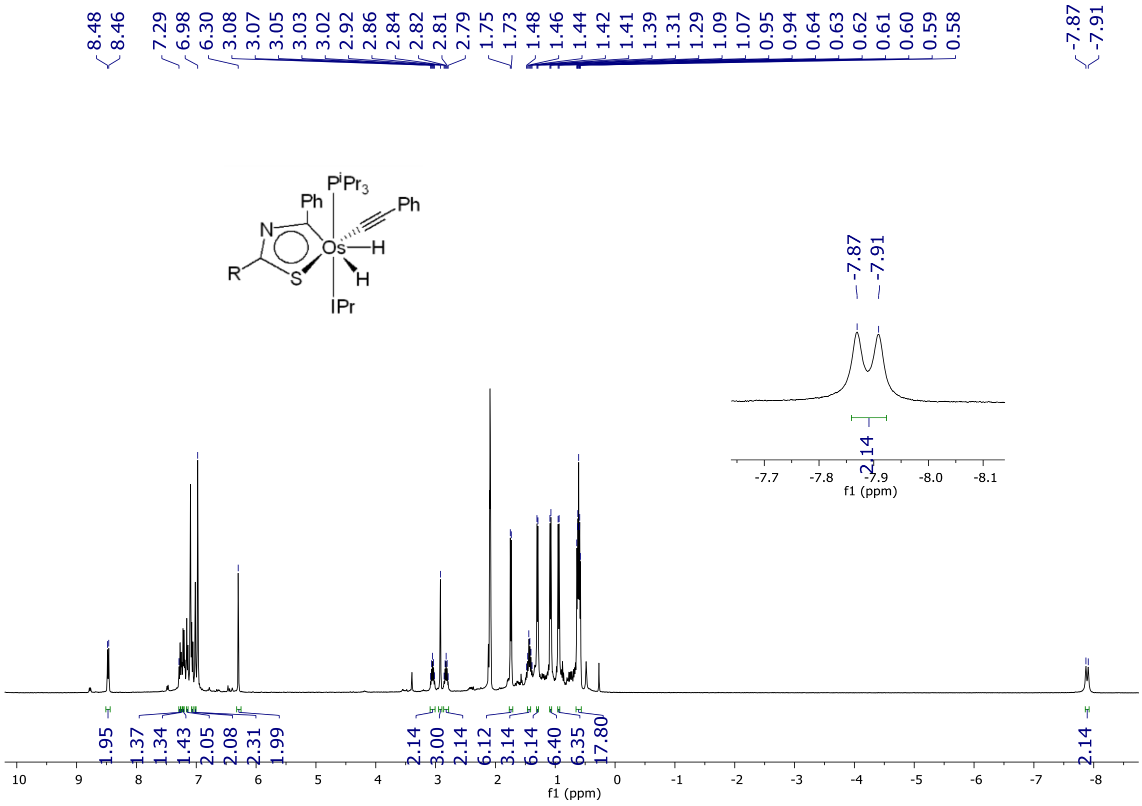

Figure S18. ^{1}H NMR spectrum (400 MHz, Tol-d_8, 298 K) of compound **7**.

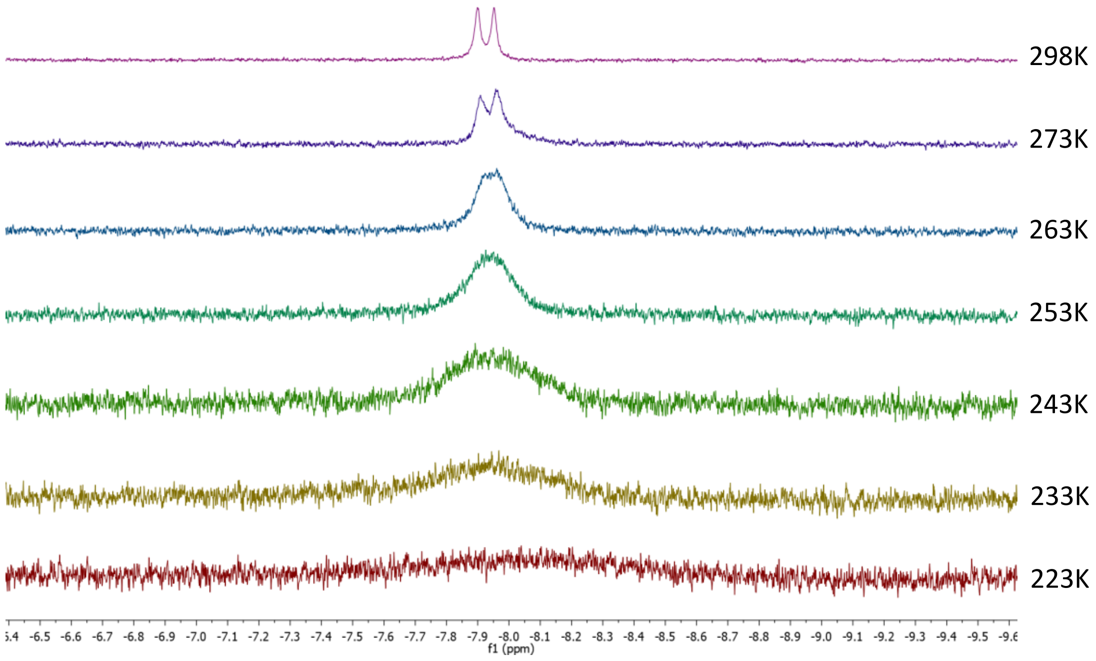

Figure S19. High field region of the ^{1}H NMR spectra (400 MHz, Tol-d_8) of compound **7** as a function of temperature.

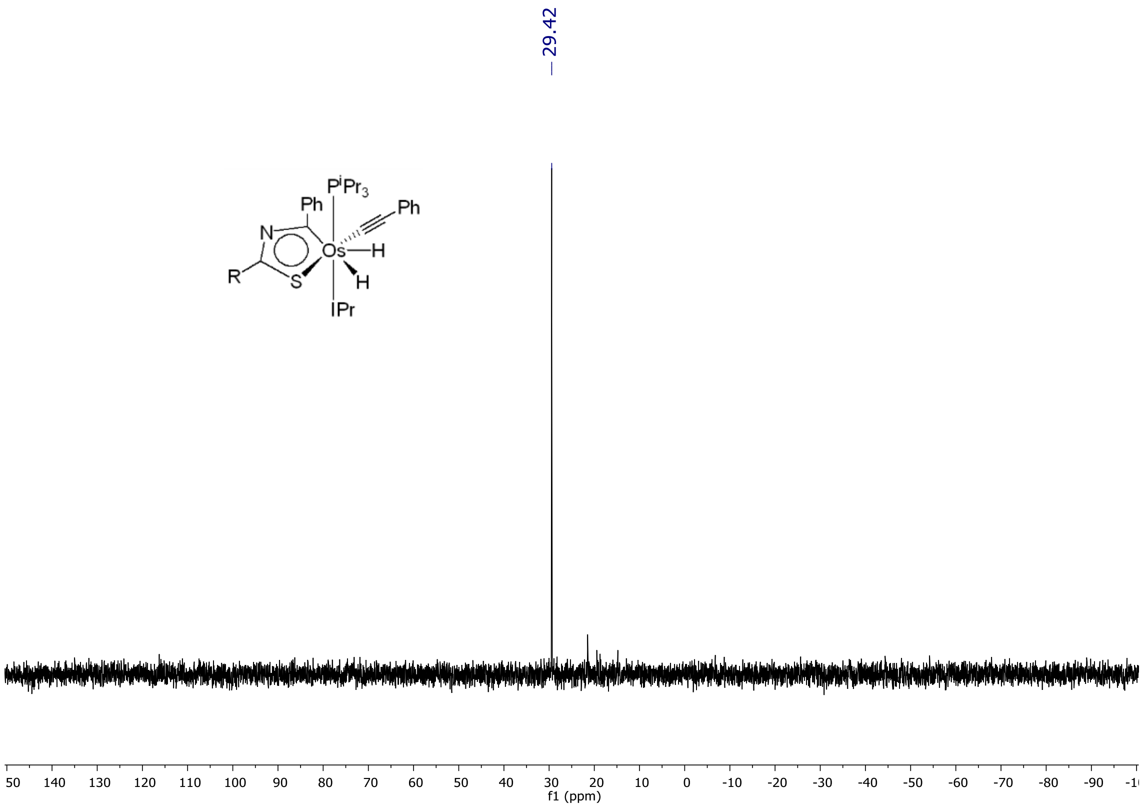

Figure S20. ^{31}P{^{1}H} spectrum (121 MHz, Tol-d_8, 298 K) of compound **7**.

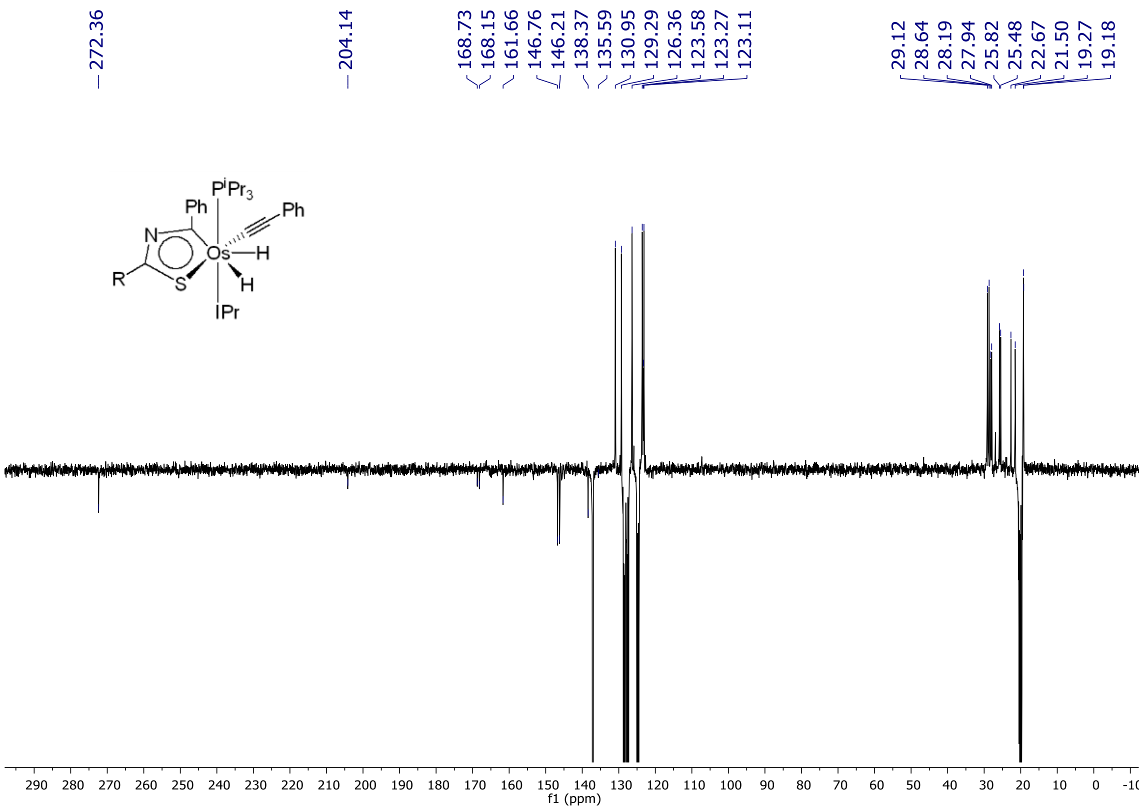

Figure S21. $^{13}C\{^1H\}$ spectrum (101 MHz, Tol-d_8, 298 K) of compound **7**.

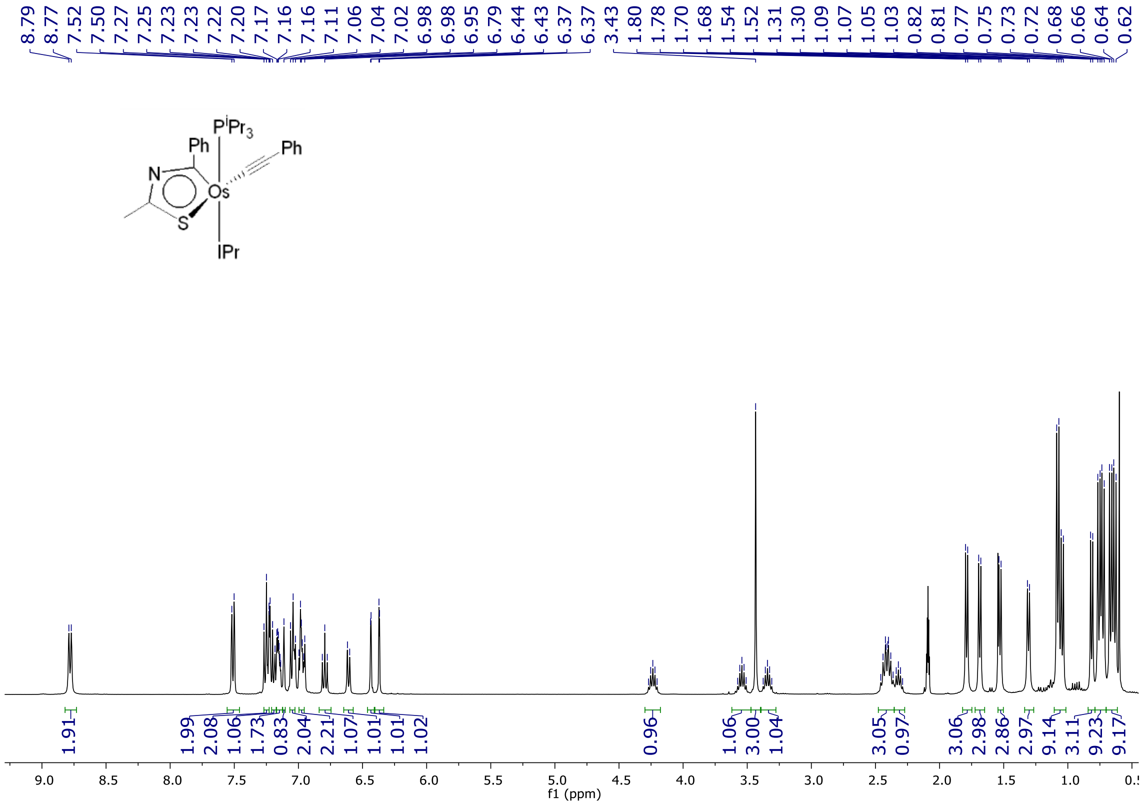

Figure S22. ^1H NMR spectrum (400 MHz, Tol-d_8, 273 K) of compound **8**.

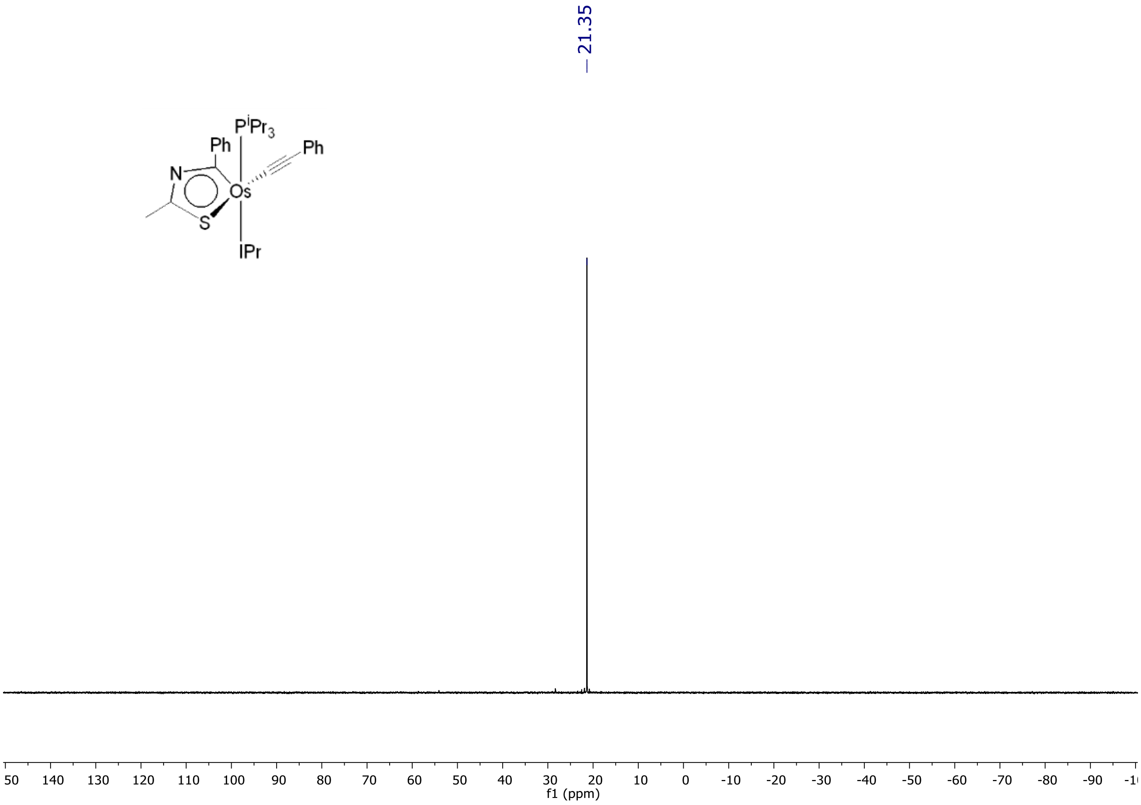

Figure S23. ^{31}P{^{1}H} spectrum (121 MHz, Tol-d_8, 298 K) of compound **8**.

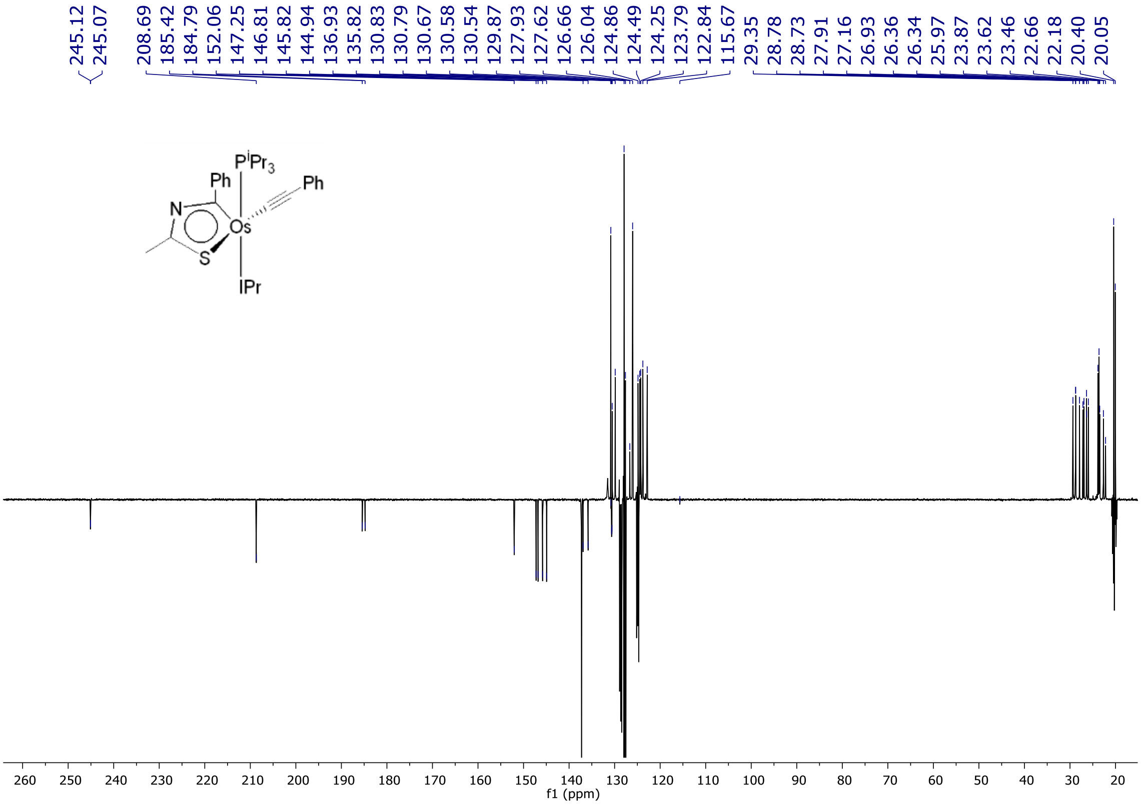

Figure S24. ^{13}C{^{1}H} spectrum (101 MHz, Tol-d_8, 273 K) of compound **8**.

- **Computational Details.**

All calculations were performed at the DFT level using the B3LYP functional[3] supplemented with the Grimme's dispersion correction D3[4] as implemented in Gaussian09.[5] Os atoms were described by means of an effective core potential SDD for the inner electron[6] and its associated double-ζ basis set for the outer ones, complemented with a set of f-polarization functions for osmium.[7] The 6-31G** basis set was used for the H, C, N, O, P and S atoms.[8] All minima were verified to have no negative frequencies. The ACID plots were performed with the AICD program.[9] The AICD plots were performed with the magnetic field vector orthogonal with respect to the ring plane and points upward (clockwise currents are diatropic). Natural bond analysis was performed with the NBO7 program.[10]

- **Energies of Optimized Structures.**

Complex **3t**

Zero-point correction=	1.083158 (Hartree/Particle)
Thermal correction to Energy=	1.144616
Thermal correction to Enthalpy=	1.145560
Thermal correction to Gibbs Free Energy=	0.991240
Sum of electronic and zero-point Energies=	-2881.604832
Sum of electronic and thermal Energies=	-2881.543374
Sum of electronic and thermal Enthalpies=	-2881.542430
Sum of electronic and thermal Free Energies=	-2881.696750

Complex 5t

Zero-point correction=	1.020662 (Hartree/Particle)
Thermal correction to Energy=	1.077817
Thermal correction to Enthalpy=	1.078761
Thermal correction to Gibbs Free Energy=	0.931548

Sum of electronic and zero-point Energies= -2748.429329
Sum of electronic and thermal Energies= -2748.372174
Sum of electronic and thermal Enthalpies= -2748.371230
Sum of electronic and thermal Free Energies= -2748.518443

Complex 8t

Zero-point correction= 1.114165 (Hartree/Particle)
Thermal correction to Energy= 1.178078
Thermal correction to Enthalpy= 1.179022
Thermal correction to Gibbs Free Energy= 1.018061
Sum of electronic and zero-point Energies= -3055.598424
Sum of electronic and thermal Energies= -3055.534511
Sum of electronic and thermal Enthalpies= -3055.533567
Sum of electronic and thermal Free Energies= -3055.694528

1,3-Thiazol

Zero-point correction= 0.055204 (Hartree/Particle)
Thermal correction to Energy= 0.059114
Thermal correction to Enthalpy= 0.060058
Thermal correction to Gibbs Free Energy= 0.028053
Sum of electronic and zero-point Energies= -568.999177
Sum of electronic and thermal Energies= -568.995267
Sum of electronic and thermal Enthalpies= -568.994323
Sum of electronic and thermal Free Energies= -569.026328

- **AICD plots:**

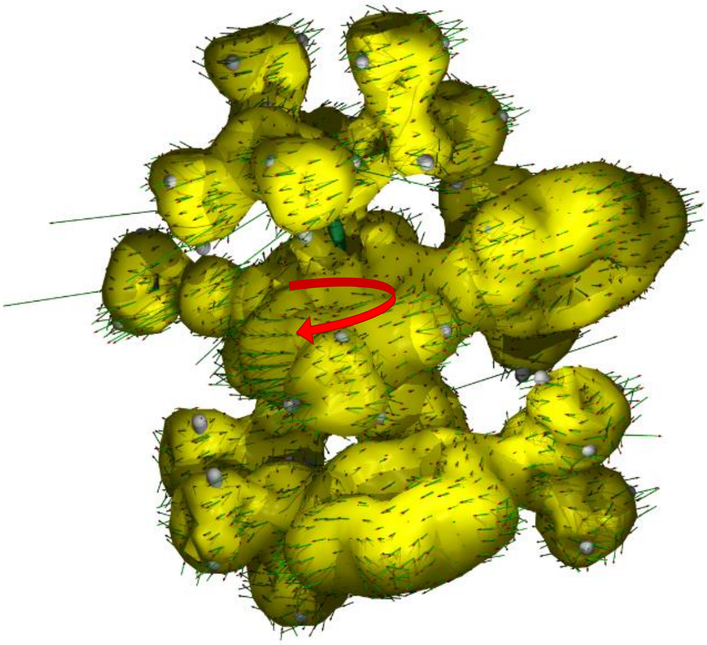

Figure S25. AICD plots of complex **3** with an isosurface of 0.02. The red arrow indicates the direction of induced current in the metalladiheterocycle.

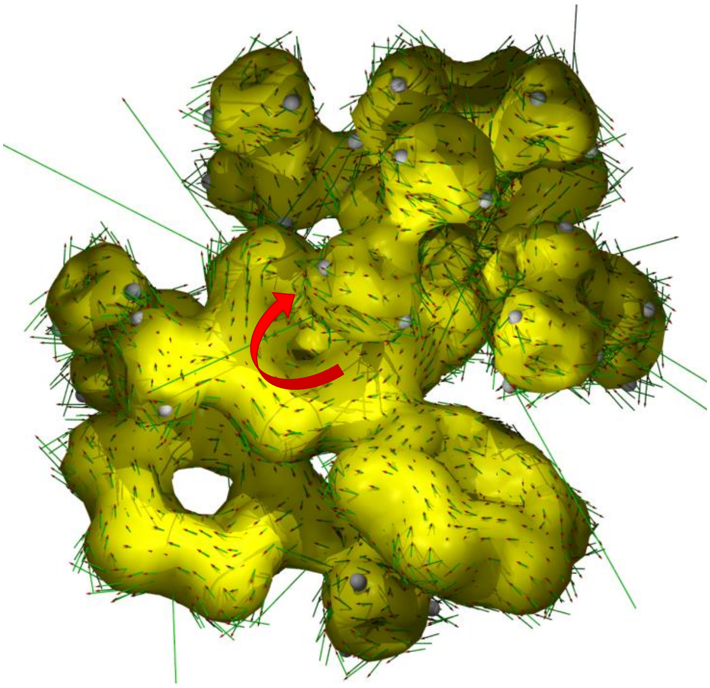

Figure S26. AICD plots of complex **5** with an isosurface of 0.02. The red arrow indicates the direction of induced current in the metalladiheterocycle.

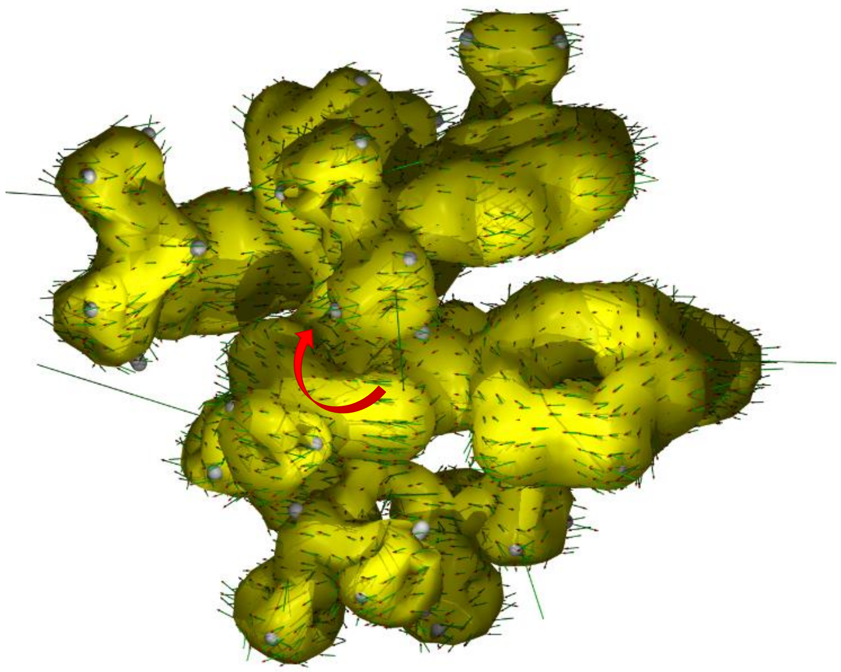

Figure S27. AICD plots of complex **8** with an isosurface of 0.02. The red arrow indicates the direction of induced current in the metalladiheterocycle.

- ## NICS and NICSzz scans:

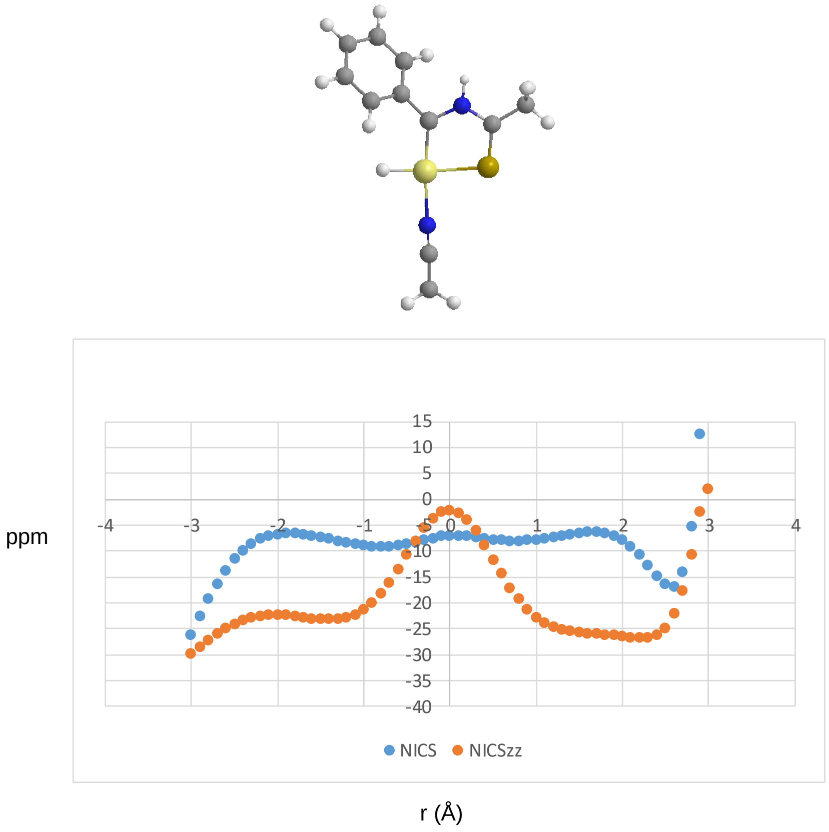

Figure S28. NICS and NICSzz scans for the diheterometallacycle of complex **3**.

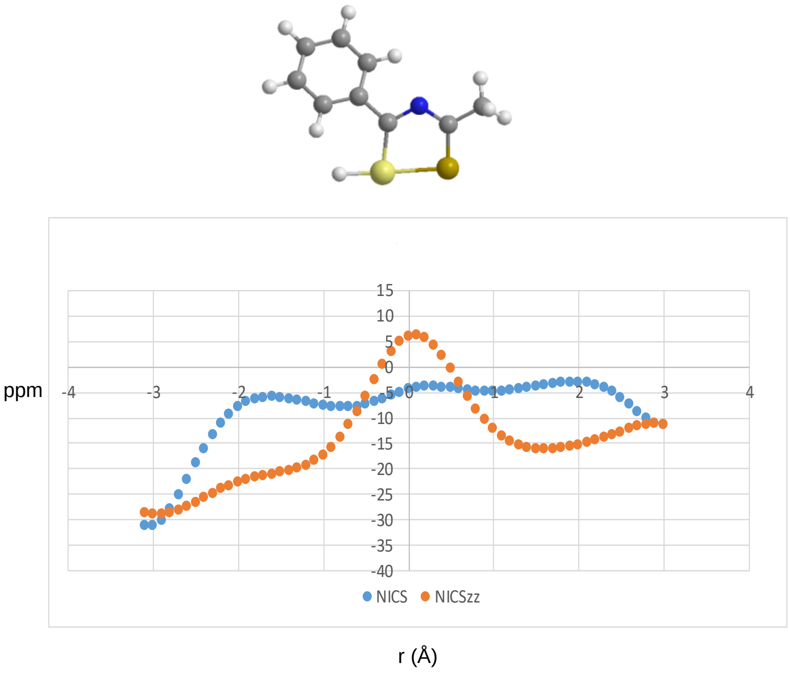

Figure S29. NICS and NICSzz scans for the diheterometallacycle of complex **5**.

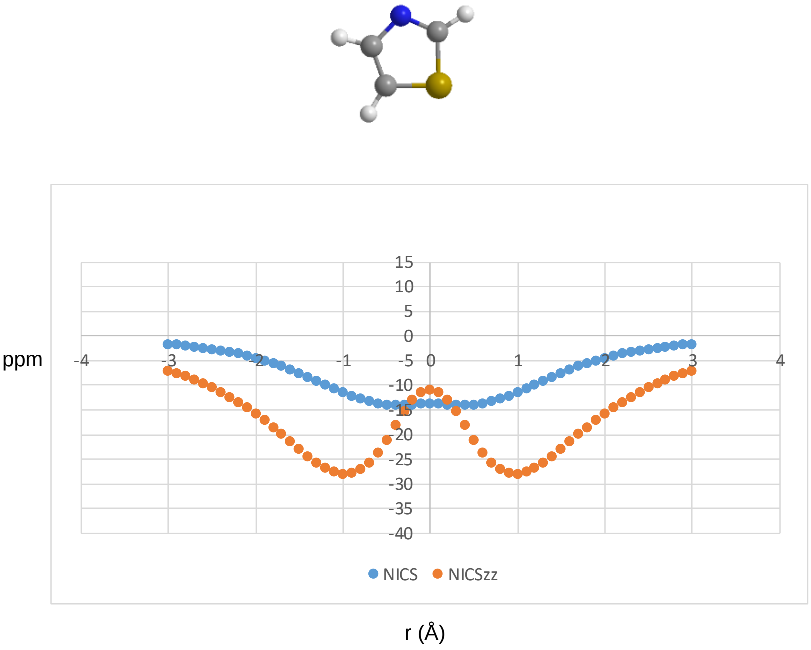

Figure S30. NICS and NICSzz scans of thiazole.

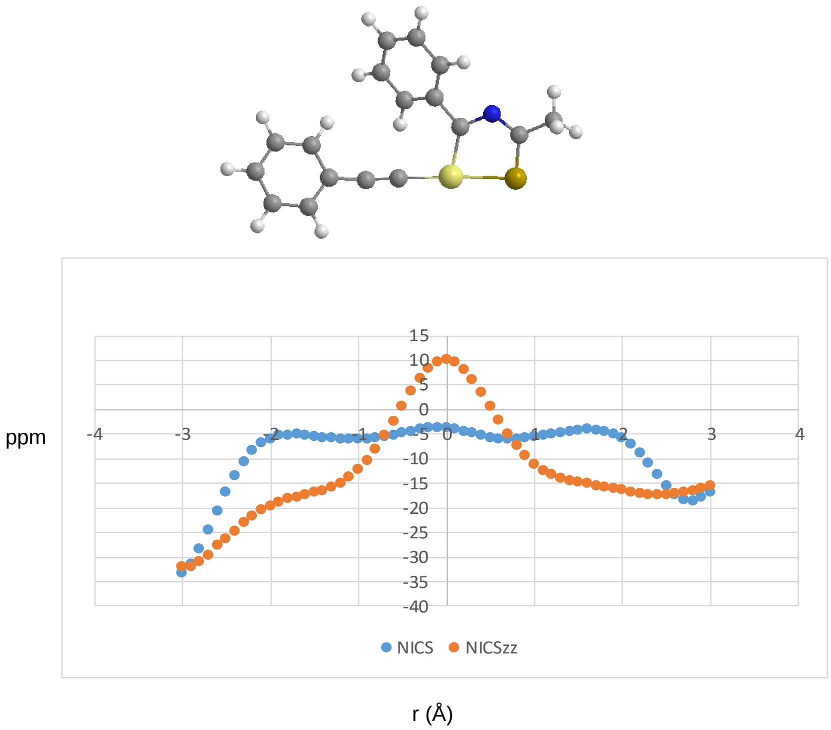

Figure S31. NICS and NICSzz scans for the diheterometallacycle of complex **8**.

- **NBO π-bond orbitals.**

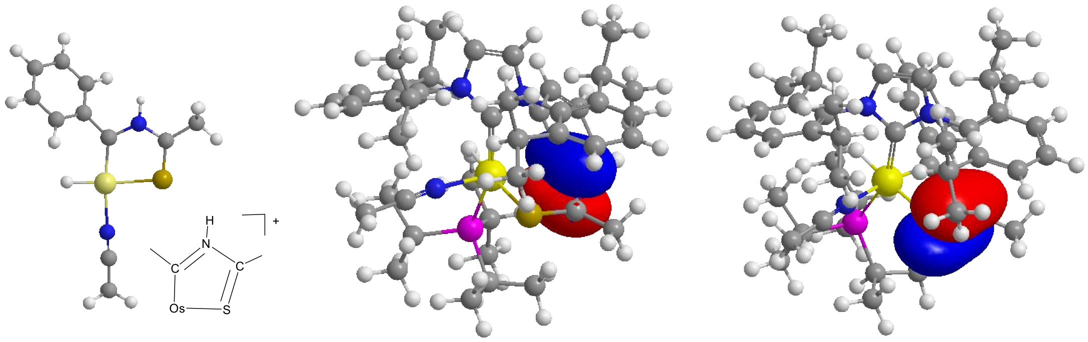

Figure S32. NBO π-bond orbitals (isovalue 0.03) in metallacycle complex **3**.

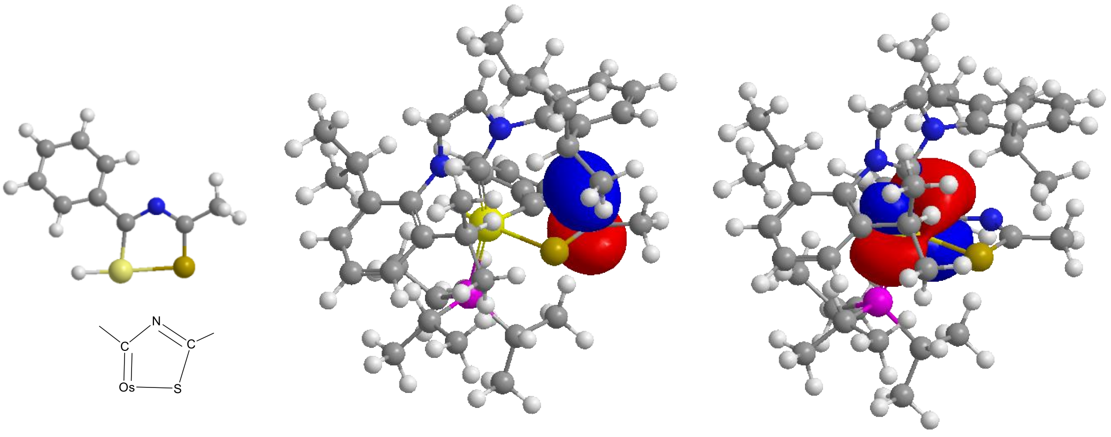

Figure S33. NBO π-bond orbitals (isovalue 0.03) in metallacycle complex **5**.

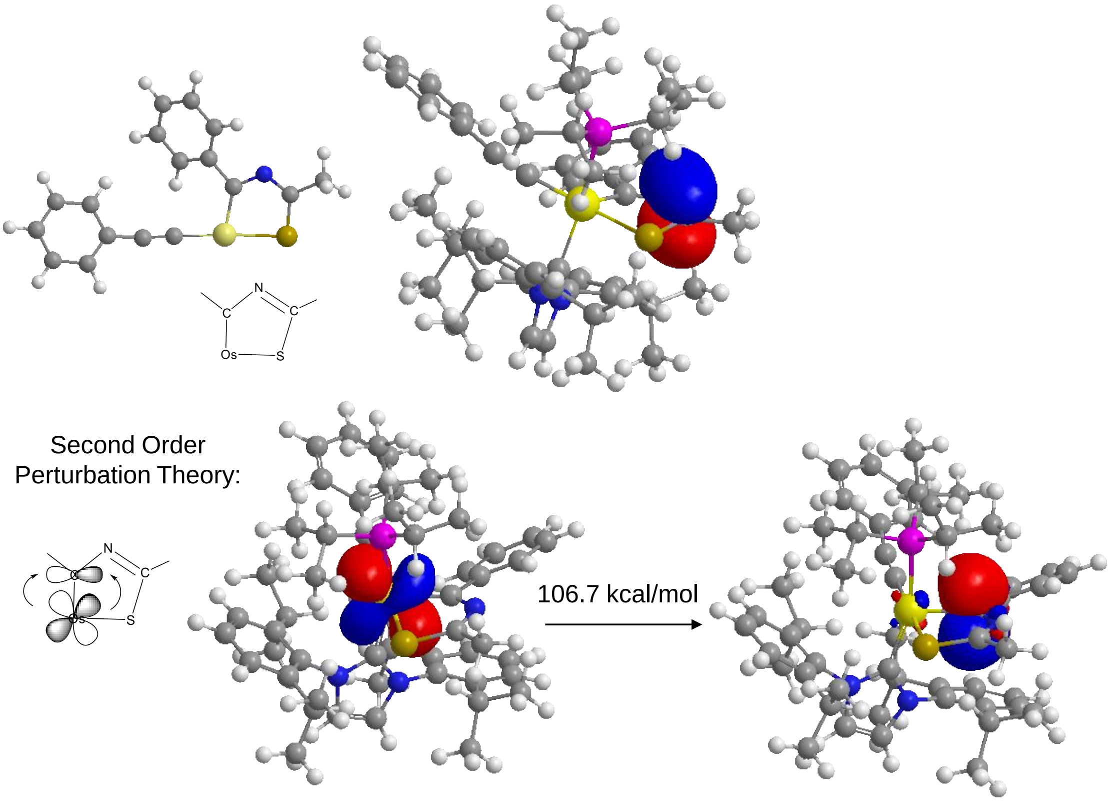

Figure S34. NBO π-bond orbitals (isovalue 0.03) in metallacycle complex **8** and pictorial view of NBO donor-aceptor interaction (isovalue 0.03).

- **X-ray Bond Lengths (Å):**

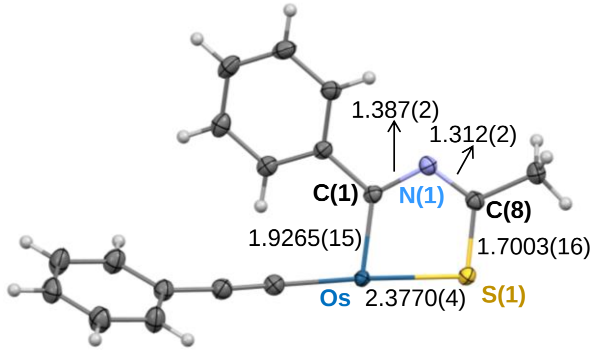

Figure S35. X-ray bond lengths in the osmathiazole ring of complex **8**.

- **Bond Wiberg (Å):**

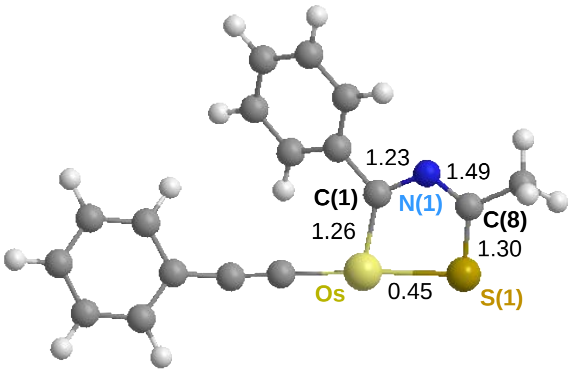

Figure S36. Bond Wiberg in the osmathiazole ring of complex **8**.

- **NBO Charges:**

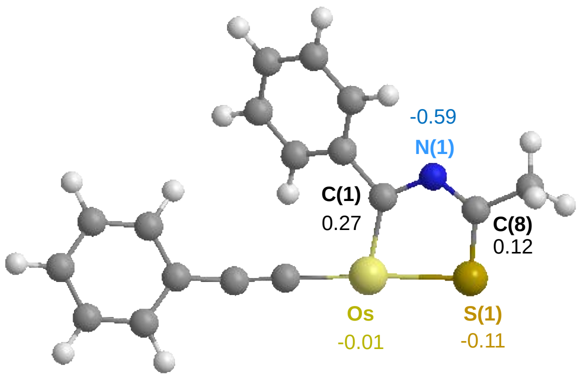

Figure S37. NBO charges in the osmathiazole ring atoms of complex **8**.

- **References.**

(1) Blessing, R. H. *Acta Crystallogr.* **1995**, *A51*, 33. SADABS: Area-detector absorption correction; Bruker- AXS, Madison, WI, 1996.

(2) SHELXL-2016/6. Sheldrick, G. M. *Acta Cryst.* **2008**, *A64*, 112-122.

(3) (a) Lee, C.; Yang, W.; Parr, R. G. Development of the Colle-Salvetti correlationenergy formula into a functional of the electron density. *Phys. Rev. B* **1988**, *37*, 785- 789. (b) Becke, A. D. Density-functional exchange-energy approximation with correct asymptotic behavior. *J. Chem. Phys.* **1993**, *98*, 5648-5652. (c) Stephens, P. J.; Devlin, F. J.; Chabalowski, C. F.; Frisch, M. J. Ab Initio Calculation of Vibrational Absorption and Circular Dichroism Spectra Using Density Functional Force Fields. *J. Phys. Chem.* **1994**, *98*, 11623-11627.

(4) Grimme, S.; Antony, J.; Ehrlich, S.; Krieg, H. A consistent and accurate ab initio parametrization of density functional dispersion correction (DFT-D) for the 94 elements H-Pu. *J. Chem. Phys.* **2010**, *132*, 154104.

(5) Gaussian 09, Revision D.01, Frisch, M. J.; Trucks, G. W.; Schlegel H. B.; Scuseria, G. E.; Robb, M. A.; Cheeseman, J. R.; Scalmani, G.; Barone, V.; Mennucci, B.; Petersson, G. A.; Nakatsuji, H.; Caricato, M.; Li, X.; Hratchian, H. P.; Izmaylov, A. F.; Bloino, J.; Zheng, G.; Sonnenberg, J. L.; Hada, M.; Ehara, M.; Toyota, K.; Fukuda, R.; Hasegawa, J.; Ishida, M.; Nakajima, T.; Honda, Y.; Kitao, O.; Nakai, H.; Vreven, T.; Montgomery, J. A.; Peralta, Jr., J. E.; Ogliaro, F.; Bearpark, M.; Heyd, J. J.; Brothers, E.; Kudin, K. N.; Staroverov, V. N.; Keith, T.; Kobayashi, R.; Normand, J.; Raghavachari, K.; Rendell, A.; Burant, J. C.; Iyengar, S. S.; Tomasi, J.; Cossi, M.; Rega, N.; S43 Millam, J. M.; Klene, M.; Knox, J. E.; Cross, J. B.; Bakken, V.; Adamo, C.; Jaramillo, J.; Gomperts, R.; Stratmann, R. E.; Yazyev, O.; Austin, A. J.; Cammi, R.;

Pomelli, C.; Ochterski, J. W.; Martin, R. L.; Morokuma, K.; Zakrzewski, V. G.; Voth, G. A.; Salvador, P.; Dannenberg, J. J.; Dapprich, S.; Daniels, A. D.; Farkas, O.; Foresman, J. B.; Ortiz, J. V.; Cioslowski, J.; Fox, D. J. Gaussian, Inc., Wallingford CT, 2013.

(6) Andrea, D.; Häußermann, U. M.; Dolg, M.; Stoll, H.; Preuss, H. Energy-adjusted ab initio pseudopotentials for the second and third row transition elements. *Theor. Chim. Acta* **1990**, *77*, 123-141.

(7) Ehlers, A. W.; Bohme, M.; Dapprich, S.; Gobbi, A.; Hollwarth, A.; Jonas, V.; Kohler, K. F.; Stegmann, R.; Veldkamp, A.; Frenking, G. A set of f-polarization functions for pseudo-potential basis sets of the transition metals SC-Cu, Y-Ag and La-Au. *Chem. Phys. Lett.* **1993**, *208*, 111-114.

(8) (a) Hehre, W. J.; Ditchfield, R.; Pople, J. A. Self-Consistent Molecular Orbital Methods. XII. Further Extensions of Gaussian-Type Basis Sets for Use in Molecular Orbital Studies of Organic Molecules. *J. Chem. Phys*. **1972**, *56*, 2257-2261. (b) Francl, M. M.; Pietro, W. J.; Hehre, W. J.; Binkley, J. S.; Gordon, M. S.; DeFrees, D. J.; Pople, J. A. Self-consistent molecular orbital methods. XXIII. A polarization-type basis set for second-row elements. *J. Chem. Phys*. **1982**, *77*, 3654-3665.

(9) (a) Geuenich, D.; Hess, K.; Köhler, F.; Herges, R. Anisotropy of the Induced Current Density (ACID), a General Method to Quantify and Visualize Electronic Delocalization. *Chem. Rev.* **2005**, *105*, 3758−3772. (b) Herges, R.; Geuenich, D. Delocalization of Electrons in Molecules. *J. Phys. Chem. A* **2001**, *105*, 3214−3220.

(10) Glendening, E. D.; Landis, C. R.; Weinhold, F. NBO 7.0: New Vistas in Localized and Delocalized Chemical Bonding Theory. *J. Comput. Chem*. **2019**, *40*, 2234–2241.
